# The cyanobacterial metabolite nocuolin a is a natural oxadiazine that triggers apoptosis in human cancer cells

**DOI:** 10.1371/journal.pone.0172850

**Published:** 2017-03-02

**Authors:** Kateřina Voráčová, Jan Hájek, Jan Mareš, Petra Urajová, Marek Kuzma, José Cheel, Andreas Villunger, Alexandra Kapuscik, Marcel Bally, Petr Novák, Martin Kabeláč, Gerhard Krumschnabel, Martin Lukeš, Ludmila Voloshko, Jiří Kopecký, Pavel Hrouzek

**Affiliations:** 1 Centre Algatech, Institute of Microbiology, The Czech Academy of Sciences (CAS) v.v.i., Třeboň, Czech Republic; 2 University of South Bohemia, Faculty of Science, České Budějovice, Czech Republic; 3 Biology Centre (CAS) v.v.i., Institute of Hydrobiology, České Budějovice, Czech Republic; 4 Centre for Phycology, Institute of Botany (CAS) v.v.i., Czech Republic; 5 Laboratory of Molecular Structure Characterization, Institute of Microbiology (CAS), Prague, Czech Republic; 6 Medical University Innsbruck, Division of Developmental Immunology, Biocenter Innsbruck, Innsbruck, Austria; 7 Tyrolean Cancer Research Institute, Innsbruck, Austria; 8 British Columbia Cancer Agency, Department of Experimental Therapeutics, Vancouver, BC Canada; 9 Saint Petersburg State University, St. Petersburg, Russia; Yong Loo Lin School of Medicine, National University of Singapore, SINGAPORE

## Abstract

Oxadiazines are heterocyclic compounds containing N-N-O or N-N-C-O system within a six membered ring. These structures have been up to now exclusively prepared via organic synthesis. Here, we report the discovery of a natural oxadiazine nocuolin A (NoA) that has a unique structure based on 1,2,3-oxadiazine. We have identified this compound in three independent cyanobacterial strains of genera *Nostoc*, *Nodularia*, and *Anabaena* and recognized the putative gene clusters for NoA biosynthesis in their genomes. Its structure was characterized using a combination of NMR, HRMS and FTIR methods. The compound was first isolated as a positive hit during screening for apoptotic inducers in crude cyanobacterial extracts. We demonstrated that NoA-induced cell death has attributes of caspase-dependent apoptosis. Moreover, NoA exhibits a potent anti-proliferative activity (0.7–4.5 μM) against several human cancer lines, with p53-mutated cell lines being even more sensitive. Since cancers bearing p53 mutations are resistant to several conventional anti-cancer drugs, NoA may offer a new scaffold for the development of drugs that have the potential to target tumor cells independent of their p53 status. As no analogous type of compound was previously described in the nature, NoA establishes a novel class of bioactive secondary metabolites.

## Introduction

Among various heterocyclic compounds found in the nature, nitrogen-oxygen heterocycles are quite uncommon. Extremely rare are reports on occurrence of heterocycles containing two nitrogen and one oxygen atom. Those are five membered oxadiazoles and six membered oxadiazines [1, 2]. One of the few cases of natural occurrence are phidianidines, low molecular indole based metabolites containing 1,2,4-oxadiazole isolated from opisthobranch mollusk *Phidiana militaris* [3]. Much larger variability of oxadiazine and oxadiazole containing structures was generated via organic synthesis; mostly with 1,2,4 or 1,3,4 arrangement containing either O-N-C-N or O-C-N-N linkage, respectively [1, 4]. Up to date there is no report on natural metabolite containing a direct N-N-O linkage in the heterocycle (1,2,3-oxadiazine or 1,2,3-oxadiazole), although compounds containing 1,2,3-oxadiazole were synthetized via organic synthesis [5–7]. Majority of above mentioned structures exhibit interesting bioactivities including potential anticancer activity [2–4, 8]. Promising results obtained especially with 1,3,4-/1,2,4-oxadiazoles and 1,2,4-oxadiazines lead to structure-activity relationship studies. In several of the synthetic derivatives, some of which have been inspired by natural products, high efficiency against cancer cells in nanomolar range was detected [2, 3]. Thus oxadiazines and oxadiazoles are considered as an important chemical platform for novel anticancer compounds [2].

One of the common features of cancers is their resistance to a broad range of cell death stimuli. Therefore, compounds that activate apoptosis [9] can have the potential for pharmaceutical leads. Classical apoptotic pathways [10, 11] initiated via death receptors [12, 13] or triggered by Bcl2 family proteins along the mitochondrial route [14] involve caspases (cysteine-aspartate proteases), which play an important role in coordinating the controlled disassembly of a cell. Irrespective of the triggering mechanism, apoptotic signals triggering either pathway usually converge at the level of effector caspases (-3, -6, and -7) [11]. Broad range of intra and/or extracellular stimuli (DNA damage, endoplasmic reticulum stress, blockage of cytoskeleton dynamics) can trigger apoptosis. In case of 1,3,4-oxadiazine containing structures several targets have been recognized–including e.g. histone deacetylase, methionine aminopeptidase and thymidylate synthetase inhibition [2]. Inhibition of these enzymes leads the cell into cell cycle arrest and subsequent apoptosis. Apoptosis has long been linked to a specific morphotype but it has become apparent that the morphological uniformity can be masked by the simultaneous induction of other cell death pathways [15].

Cyanobacteria are well-known to produce diverse natural products. The majority of reported compounds can be classified as low-molecular-weight heterocyclic derivatives, polyketides, macrolactones, cyclic or linear oligopeptides, and lipopeptides [16–18]. The biosynthesis of these metabolites is in most cases performed by the equally diverse polyketide synthetases (PKS) and non-ribosomal peptide synthetases (NRPS) [16,17]. Unusual structure predisposes many cyanobacterial secondary metabolites to interact with various cellular targets, which results in their high pharmacological potential.

Extensive screening for apoptosis inducers in cyanobacterial extracts, performed in our laboratory, led to the identification of possible hits in *Nostoc* sp. CCAP 1453/38 and *Nodularia* sp. HBU26. The following activity-guided fractionation of the extract of *Nostoc* sp. CCAP 1453/38 resulted in the discovery of a candidate compound, nocuolin A (NoA), exhibiting pro-apoptotic properties. In the present study, we elucidate the highly unusual structure of NoA, show its anticancer potential in a panel of human cancer cell lines and demonstrate that NoA is causing caspase-dependent apoptosis. We further report the putative NoA biosynthetic gene cluster in three cultured NoA-producing cyanobacterial strains.

## Material and methods

### Cultivation of cyanobacterial strains

The cyanobacterial strains *Nostoc* sp. CCAP 1453/38 and *Nodularia* sp. HBU26 were cultivated in BG-11 medium [19] at 28°C and light intensity of 100 μE in 15 L bioreactor bubbled with air enriched with 1.5% CO_2_. The biomass for isolation of ^15^N isotopically substituted nocuolin A was cultivated in the same cultivation conditions in BG-11 medium supplemented with Na^15^NO_3_ (Sigma Aldrich). The axenic strain of *Anabaena* sp. PCC 7108 was obtained from a PCC collection and cultured in Allen Arnon [20] and BG-11 medium [19] at 20°C and light intensity of 100 μE.

### Extraction and fractionation

The biomass was harvested by centrifugation and stored at -70°C prior to lyophilisation. Freeze-dried biomass of *Nostoc* sp. CCAP 1453/38 (13.5 g) was homogenized with sea-sand and extracted twice with 70% methanol (250 mL). Extracts were combined and methanol was evaporated under reduced pressure at 30°C. The resulting aqueous extract (160 mL) was loaded onto a C_18_-SPE cartridge (Supelco, 20 mL, 5 g). The cartridge was gradually eluted with 20 mL of water, n-hexane, dichloromethane, acetonitrile, methanol, and methanol containing 1% hydrochloric acid. The target compound was present in the dichloromethane fraction. Subsequently, the solvent was evaporated until dryness. The dry residue was dissolved in methanol, filtered through the polytetrafluoerthylene (PTFE) 0.45 μm filter and permeated through a Sephadex LH-20 column (290 × 30 mm) using methanol as the mobile phase at a flow rate of 0.33 mL.m^-1^. The presence of NoA in the Sephadex LH-20 fractions was determined by HPLC-HRMS (see below). Fractions containing NoA were pooled and concentrated under vacuum. The solid residue was dissolved in MeOH and fractionated by high performance countercurrent chromatography (HPCCC). The selected solvent system for HPCCC fractionation (n-hexane:ethyl acetate:methanol:water, 8:2:5:5, v/v/v) was prepared by vigorous mixing of the corresponding portions of solvents. The phases were left to equilibrate overnight and separated using a separating funnel. Shortly before their use, the phases were degassed by sonication. The sample solution was prepared by dissolving the NoA fraction separated by Sephadex in 3 mL of the lower phase of the solvent system. The resulting sample solution was filtered through a 0.45 μm membrane before use. The lower phase of the solvent system was used as the mobile phase and the upper phase as the stationary phase. The HPCCC column was initially filled with the upper phase (stationary phase). The apparatus was then rotated at 1,200 rpm, and the lower phase (mobile phase) was pumped into the column at a flow rate of 2 mL.m^-1^. The temperature of the apparatus was set at 28°C. After the mobile phase front emerged and hydrodynamic equilibrium was established, the NoA fraction was injected through the sample injection valve. The effluent from the outlet was continuously monitored at 250 nm and collected according to the response of the detector for subsequent off-line HPLC-HRESI-MS/MS analysis. NoA was identified in the HPCCC fraction eluted at a retention time between 90 and 110 m. The NoA enriched fraction obtained by HPCCC was concentrated under vacuum at 30°C and then subjected to semi-preparative high performance liquid chromatography (HPLC). As the first step C_18_ column (Watrex Reprosil C_18_, 250 × 8 mm, 5 μm) was used with a H_2_O/methanol gradient elution (45/55 (0 m), 0/100 (20 m), 0/100 (25 m). NoA eluted at retention time of 21 m. Obtained NoA fraction was further re-purified on a phenyl column (Watrex Reprosil 100 Phenyl, 250 × 8 mm, 5 μm) using water and methanol as a mobile phase at a flow rate of 3 mL.m^-1^. The gradient was as follows: H_2_O/methanol 40/60 (0 m), 20/80 (15 m), 20/80 (20 m), 0/100 (25 m), 0/100 (30 m). The fraction of NoA collected at 17.8 m and evaporated under nitrogen affording 0.67 mg of NoA at 99% purity (determined by HPLC-MS). The yield of NoA with respect to dry cyanobacterial biomass was 0.005%. Isotopically substituted NoA was isolated using identical protocol performed on 10 g of freeze-dried biomass.

### Liquid chromatography-mass spectrometry

*Nostoc* sp. CCAP 1453/38 and *Anabaena* sp. PCC 7108 70% methanolic extracts were analysed on Thermo Scientific Dionex UltiMate 3000 UHPLC (Thermo Scientific) equipped with a diode array detector (DAD) and high resolution mass spectrometry with electrospray ionization source (ESI-HRMS; Impact HD Mass Spectrometer, Bruker). HPLC separation was performed on a reversed phase Kinetex Phenomenex C_18_ column (150 x 4.6 mm, 2.6 μm) with H_2_O/acetonitrile acidified with 0.1% HCOOH as a mobile phase. Flow rate during analysis was 0.5 mL.m^-1^. The gradient was as follows: H_2_O/acetonitrile 85/15 (0 m), 85/15 (in 1 m), 0/100 (in 20 m), 0/100 (in 25 m) and 85/15 (in 30 m). The HPLC was connected to a mass spectrometer with the following settings: dry temperature 200°C; drying gas flow 12 L.m^-1^; nebulizer 3 bar; capillary voltage 4500 V; endplate offset 500 V. The spectra were collected in the range 20–2000 m/z with spectra rate 3 Hz. Calibration was performed using LockMass 622 as internal calibration solution and CH_3_COONa at the beginning of each analysis. For experiments with CD_3_OD, a direct infusion with a flow of 30 μL.m^-1^ was performed. A small amount of pure NoA was dissolved in 100 μL CD_3_OD containing 0.05% HCOOH. Drying gas flow and nebulizer were set to 10 L.m^-1^ and 2 bar, respectively. Other settings remained unchanged. Collision energy for MS/MS spectra was set to 20 eV. Fragmentation experiments were repeated at a high resolution mass spectrometer with Fourier transformation—Solarix XR (Bruker) to obtain better exact mass values. The settings were the following: dry temperature 200°C; drying gas flow 12 L.m^-1^; nebulizer 3 bar; capillary voltage 4500 V; endplate offset 500 V. The spectra were collected in the range 20–2000 m/z with spectra rate 3 Hz.

### NMR measurement

NMR spectra were recorded on a Bruker Avance III 600 MHz spectrometer equipped with TCI CryoProbe (600.23 MHz for ^1^H, 150.93 MHz for ^13^C, 60.82 ppm for ^15^N, Bruker Biospin GmbH, Rheinstetten, Germany) and a Bruker Avance III 700 MHz spectrometer equipped with TCI CryoProbe (700.13 MHz for ^1^H, 176.05 MHz for ^13^C, 70.94 ppm for ^15^N, Bruker Biospin GmbH, Rheinstetten, Germany) in CD_3_CN at 303.2 K. The residual solvent signals were used as an internal standard (*δ*_H_ 1.930 ppm and *δ*_C_ 1.39 ppm). ^1^H NMR, ^13^C NMR, COSY, ^1^H-^13^C HSQC, ^1^H-^13^C HMBC, ^1^H-^15^N HMBC, and ^1^H-^13^C HSQC-TOCSY spectra were measured using the standard manufacturer’s software. The ^1^H NMR spectrum was zero filled to 2-fold data points and multiplied by a window function (two-parameter double-exponential Lorentz-Gauss function) before Fourier transformation to improve the resolution. The ^13^C NMR spectrum was zero filled to 2-fold data points. Subsequently, line broadening (1 Hz) was used to improve signal-to-noise ratio. Protons were assigned by COSY and ^1^H-^13^C HSQC-TOCSY, and the assignment was transferred to carbons by HSQC. The chemical shifts are given in the *δ* scale (ppm) and coupling constants are given in Hz. The digital resolution allowed us to present the proton and carbon chemical shifts to three and two decimal places, respectively. The proton chemical shift readouts from HSQC are reported to two decimal places.

### Fourier transform infrared spectroscopy (FTIR)

2 μL of NoA sample in methanol solution (8 mg.mL^-1^) were spread on a single well of Si 384 well plate. Pure methanol was used as a blank for background subtraction. Samples were measured on Nicolet IS10 spectrometer equipped with Microarray reader compartment equipped with DTGS detector (Deuterated Tri Glycine Sulphate detector). The compartment with the detector was continuously flushed with dry nitrogen to avoid moisture. Absorbance spectra were collected between 400 cm^-1^ and 4000 cm^-1^ at a spectral resolution of 4 cm^-1^ and 64 scans were co-added and averaged. A Blackman-Harris three-term apodization function was used, with a zero-filling factor of 2. Spectra were baseline corrected using the rubber-band method.

### Computational chemistry methods

Rough possible 3D structures of NoA and their conformers were created in ACD/ChemSketch 12 software. Several possible NoA structures were optimized by the Hartree-Fock method with 6-31G basis sets. All the calculations were performed using Gaussian 09 program package running at computational clusters of MetaCentrum (http://www.metacentrum.cz). IR spectra were obtained by a harmonical vibrational analysis using the scaling factor 0.903 from Computational Chemistry Comparison and Benchmark Database [21] was applied systematically on all normal vibrational modes. Calculated absorption was converted to T(%) using the formula: T(%) = 100*10^-x^, where x = A/Amax. To obtain a better agreement between the calculated and measured spectra, especially in the fingerprint region, the non-solvated dimeric form of NoA as well as the NoA adducts with solvents used in HPLC purification (methanol, water) were taken into account.

### Molecular and bioinformatic analysis

Single filaments of *Nostoc* sp. CCAP 1453/38 and *Nodularia* sp. HBU26 were isolated for whole genome amplification (WGA) and subsequent preparation of a whole genome sequencing (WGS) library, as described previously [22]. Briefly, glass capillary technique was applied to isolate the filaments excluding minor bacterial contaminants. A set of 20 filaments was then used as a template for WGA. Multiple displacement amplification (MDA) using Repli-g Mini Kit (Qiagen) was followed by PCR and sequencing tests for the content of cyanobacterial 16S rRNA gene using primers 16S387F and 16S1494R [23]. Positive samples (14 and 8 MDA products yielding clear 16S rRNA gene sequences of *Nostoc* and *Nodularia*, respectively) were then pooled to create a template for WGS. The DNA was sent for commercial *de novo* genome sequencing (EMBL Genomics Core Facility, Heidelberg, Germany) using Illumina MiSeq (Illumina) with a ~350-bp average insert length Pair-End library and 250-bp reads (approximately 1.4 Gbp data yield). The sequence data were deposited in the NIH Sequence Read Archive (http://trace.ncbi.nlm.nih.gov/Traces/sra/) and they are accessible under the NCBI BioProjects PRJNA266493 and PRJNA371726, BioSamples SRS738301 and SRS1963306. Raw data from *de novo* WGS were assembled using CLC Bio Genomics Workbench v. 7.5 (CLC Bio). The genomic scaffolds were loaded into Geneious Pro R9 (Biomatters; available from http://www.geneious.com) and investigated for NRPS, PKS, and fatty acyl-AMP ligase (FAAL) genes using *blastp* searches with several known cyanobacterial amino acid adenylation (A-domains), ketosynthetase (KS) and FAAL domains as queries. Contigs yielding high similarity hits (E-value < 10^−20^) were then analyzed using the Glimmer 3 [24] algorithm to discover putative ORFs. Functional annotation of the ORFs (S1 Table) was conducted by applying a combination of *blastp*/CDD searches against the NCBI database, and by antiSMASH secondary metabolite gene cluster annotation pipeline [25]. The complete putative biosynthetic pathway for NoA from *Nostoc* sp. CCAP 1453/38 and *Nodularia* sp. HBU26 were uploaded to the NCBI GenBank database under accession numbers KP143720 and KY594676, respectively. The putative *noc* gene clusters in *Anabaena* sp. PCC 7108 and *Trichormus* sp. NMC-1 were identified during the *blastp* analyses of the *Nostoc* candidate NoA cluster, and subsequently processed using the same methods.

### Isotopically substituted tryptophan/phenylalanine feeding

The culture of *Nostoc* sp. CCAP 1453/38 was pre-grown in Allen-Arnon medium [20] at 28°C and light intensity of 100 μE in 500 mL glass tube bubbled with air enriched by 1.5% CO_2_. Part of the liquid culture (100 mL) was supplemented with L-Tryptophan– ^15^N_2_ (Sigma Aldrich, 574600) to obtain final concentration of 0.1 mg.mL^-1^ and L-Phenylanaline– ^13^C_9_^15^N_2_ (Sigma Aldrich, 608017). Control cultures enriched by the same amounts of non-substituted Trp and Phe were grown for 3 days, harvested by centrifugation and kept in -70°C prior to lyophilisation.

### Cell line cultures

HeLa cells used for CellTiter-Glo® Luminescent Cell Viability Assay and Caspase-Glo® 3/7 assay were kindly provided by Prof. Jan Kopecký (Institute of Parasitology, České Budějovice, Czech Republic) and were cultured in RPMI 1640 media supplemented with 10% FBS, 2mM L-glutamine and 1% antibiotics. HeLa S3 cells used for FACS analyses and immunoblotting were kindly provided by Prof. Andreas Villunger (Medical University Innsbruck, Division of Developmental Immunology, Biocenter Innsbruck, Austria) and were cultured in DMEM with 10% FBS and 2mM L-glutamine and 1% antibiotics. Cancer cell lines used for IC_50_ evaluation: A549^*1*^, H460^*1*^, BxPC3^*1*^, MCF7^*1*^, JIMT1^*2*^, U87^*2*^, U251^*2*^, SKBR3^*3*^ and OVCAR5 were purchased from American Type Culture Collection (Manassas, VA, USA). Cell lines^*1*,*2*,*3*^ were cultured in the following media: RPMI1640^*1*^, DMEM^*2*^, McCoy's 5A^*3*^ supplemented with 10% FBS and 2mM L-glutamine, the OVCAR5 cells were cultured in RPMI 1640 plus 20% FBS, 2mM L-glutamine, 10 μg.mL^-1^ insulin and 10mM HEPES.

### Viability measurements for IC_50_

Cells were seeded onto a 384-well flat bottom plate and allowed to adhere overnight under normal growth conditions. Serial dilutions of NoA compound were then added to the wells (each dilution in triplicate) and cells were grown for the next 72 hours. To assess cell viability we used two end-point methods. First, metabolic activity was determined with PrestoBlue® Cell Viability Reagent (according to the manufacturer’s instructions, ♯A13261, Life technologies). The fluorescence was measured using the FLUOstar OPTIMA plate reader (BMG Labtechnologies, Germany). For IC_50_ calculation, the background media fluorescence was subtracted from all samples. As the second method we used fluorescence microscopy to discriminate live/death cells. The cells were stained with Hoechst 33342 (Life technologies) and Ethidium homodimer (ETH, Molecular Probes, Invitrogen) followed by imaging with IN Cell 1000 Analyzer (GE Healthcare). Six to nine images per one well were acquired with 10× magnification; images were analyzed with IN Cell 1000 Investigator and/or Analyzer software. The data were reported as a percentage of viable cells normalized to control.

### IC_50_ evaluation

Data for IC_50_ evaluation were calculated using non-linear regression analysis (log agonist vs. response sigmoidal function), in GraphPad Software. The IC_50_ values were established from the fraction affected NoA-treated cells to untreated control (the mean and standard deviation were calculated out of three independent experiments).

### Caspase 3/7 homogeneous assay

HeLa cells were plated onto a 96-well flat bottom white plate one day prior to the treatment (1×10^4^ cells/well). The assay was performed according to the manufacturer’s instructions (Caspase-Glo® 3/7 Assay, ♯G8091, Promega): equal volume of the reagent (100 μl) was added to each well and let to incubate for 30 m, the luminescent signal (RLU) was measured using Tecan Infinite 200 reader with integration time of 1s. Simultaneously, we monitored the cell division rate by time-lapse microscopy experiment (Zeiss Axio Observer Z.1 equipped with cultivation chamber for CO_2_ regulation and temperature control) in an extra plate (96-well flat bottom transparent culture plate), which was prepared with the same settings as described above. The number of cells was counted in a section of the microscopic view (700 × 350 μM) manually in AxioVision software. The number of cells present in the well at the time of caspase-3/7 activity measurement was obtained by multiplying the initial cell number seeded in the well by the number of cell divisions at a particular time. Three independent experiments were performed (each experiment was done in triplicates); the acquired values (averages of the triplicates) were normalized to the number of cells. Taxol (1 μM; ♯T7402, Sigma) and Staurosporine (1 μM; ♯S5921, Sigma) were used as positive controls. Z-VAD-FMK (10 μM; ♯FMK001, R&D Systems) was used as the pan caspase inhibitor and was added one hour prior to NoA-treatment.

### Immunoblotting

Harvested HeLa cells were lysed in lysis-buffer (10 mM HEPES, 1.5 mM MgCl_2_, 300 mM sucrose, 10 mM KCl, 0.5% NP40, Roche Complete Protease Inhibitor Cocktail (Roche, Vienna, Austria), 5 mM DTT prepared freshly from a 1 M stock) on ice for at least one hour. Proteins were separated by SDS/PAGE, transferred to a nitrocellulose membrane and incubated with the relevant antibodies: cleaved Caspase-3 ([Asp175] Cell Signalling), Parp (#9542, Cell Signalling), Hsp 90 α/β (sc-13119, Santa Cruz Biotechnology). Treatments: NoA compound (6.7 μM) for 12/24/36/48 h and Staurosporine 1 μM for 4 h (♯S5921, Sigma), co-treatment: pan-caspase inhibitor Q-VD-OPh (10 μM, ♯SML0063, Sigma) was added one hour before.

### The ATP content in time course experiment

HeLa cells were plated onto a 96-well flat bottom white tissue culture plate (1×10^4^ cells/well) and allowed to adhere overnight. The ATP cellular level was quantified using the CellTiter-Glo® Luminescent Cell Viability Assay (♯G7570, Promega, according to the manufacturer’s instructions). The luminescence signal was measured with Tecan Infinite 200 reader and integration time of 1s. Data were acquired as technical triplicates in three independent experiments. The data were expressed in relative luminescent units (RLU): the luminescence values of treated and untreated cells were normalized to the initial values of control; each time point is a mean of normalized measurements and its standard deviation (SD).

### Quantification of cell death, sub-G1

Harvested HeLa cells were fixed in pre-chilled 70% ethanol/PBS and kept at –20°C at least overnight. For sub-G1 analysis cells were washed twice with PBS, followed with RNase A (Sigma) digestion (100 μg.mL^-1^ in PBS, 30 m at 37°C), after which the cells were stained with propidium iodide (40 μg.mL^-1^) and analysed by flow cytometry BD FACSCalibur^TM^.

## Results

### NoA structure elucidation

**Fig 1:**
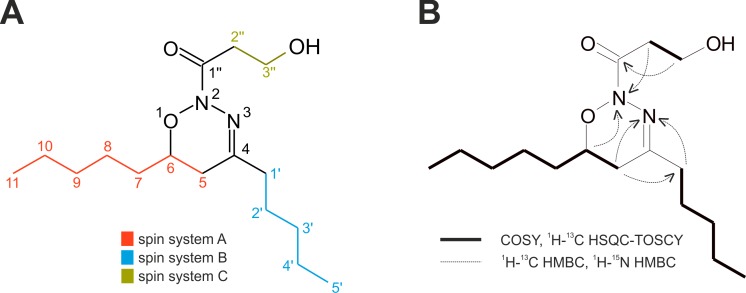
Chemical structure of nocuolin A. Panel (A) demonstrates nocuolin A molecule with highlighted spin systems A, B, and C as obtained by NMR—COSY and ^1^H-^13^C HSQC-TOSCY. Panel (B) displays the crucial NMR correlations. See also S1–S12 Figs.High resolution mass spectrometry (HRMS) measurement of NoA provided ions corresponding to a protonated molecule [M+H]^+^, sodium adduct [M+Na]^+^, potassium adduct [M+K]^+^, and sodium adduct of a dimer [2M+Na]^+^ (S2 Fig), which allowed us to determine the exact mass of the protonated molecule (299.2233) and to calculate the neutral formula to C_16_H_30_N_2_O_3_ with high accuracy (∆ 1.2 ppm in FTMS). In parallel we have found production of this compound in extracts of two other cyanobacterial strains, *Anabaena* sp. PCC 7108 (axenic strain) and *Nodularia* sp. HBU26, via HPLC-HRMS/MS measurements (S1B and S1C Fig). The analysis revealed the occurrence of a molecular ion perfectly matching that of NoA in molecular mass, fragmentation pattern and retention behaviour in extracts of both strains (S1 Fig). However, due to the extremely low level of NoA production in PCC 7108 and HBU26 we have performed the structure elucidation on the compound purified from strain *Nostoc* sp. CCAP 1453/38.

We identified NoA (Fig 1) as a cytotoxic constituent in the crude *Nostoc* sp. CCAP 1453/38 extract using HPLC activity-guided fractionation (S1 Fig). The compound is a minor component of the *Nostoc* sp. CCAP 1453/38 biomass (average content of 0.05 ‰ (w/w)).

The presence of two nitrogen atoms in the molecule was proved by growing the cyanobacterium with isotopically substituted Na^15^NO_3_ and a subsequent increase of the protonated molecule mass by 2 Da as recorded in the HRMS spectrum (S2 Fig). Furthermore, the presence of one exchangeable hydrogen atom was demonstrated by a 1 Da increase in [M+H]^+^, [M+Na]^+^ and [M+K]^+^ and a 2 Da increase in [2M+H]^+^ and [2M+Na]^+^ in HRMS experiments using deuterated methanol as a solvent. The ^13^C NMR spectrum contained 15 signals (Table 1 and S3 Fig), which in fact represented 16 carbons due to the overlap of two methyls confirmed by the ^1^H-^13^C HSQC experiment (S4 Fig). Their distribution was the following: two methyls, eleven methylenes, one methine and two quaternary carbons. The ^1^H NMR spectrum (S5 Fig) contained three isolated spin systems, which were identified in COSY (S6 Fig), ^1^H-^13^C HSQC-TOCSY, and ^1^H-^13^C HMBC (S7 Fig) as follows: CH_3_-CH_2_-CH_2_-CH_2_-CH_2_- (spin system A), CH_3_-CH_2_-CH_2_-CH_2_-CH_2_-CH(R^1^)-CH_2_- (spin system B), and -CH_2_-CH_2_-R^2^H (spin system C) (Fig 1). The spin systems A and B are interconnected via attachment to one quaternary carbon (carbon shift 152.09 ppm) as approved by the ^1^H-^13^C HMBC spectrum (S7 Fig). Their connection was further confirmed by the HMBC correlation observed between the terminal C-5 carbon and the H1’ hydrogen and *vice versa* C-1’ to H-5 (Fig 1). According to the ^1^H-^15^N HMBC spectrum, both the H-5 and the H-1’ hydrogens have also contact to a nitrogen (302 ppm), which proves the bond between the quaternary carbon C-4 and nitrogen N-3. Moreover, this nitrogen is directly bonded to the second nitrogen atom N2 with a chemical shift of 222 ppm (S7 Fig). This connection was confirmed by the observation of splitting the ^1^H-^15^N HMBC spectrum in the F1 dimension (S8 Fig), which is caused by coupling between the two nitrogen atoms in the molecule. The coupling constant (^1^JNN = 17 Hz) agrees with the previously reported one-bond couplings between nitrogens [26–28]. The nitrogen atom N-2 shows correlation with the H-6 hydrogen belonging to the spin system A, which integrates the six-membered heterocyclic core of NoA.

**Table 1:** NMR data for nocuolin A. The ^1^H, ^13^C, and ^15^N NMR data are presented (600.23 MHz for ^1^H, 150.93 MHz for ^13^C, 60.82 ppm for ^15^N, CD_3_CN, 303.2 K). ^15^N NMR of ^15^N labelled sample (70.93 MHz, CD_3_CN, 293.2 K) *δ* 222, 302. Asterisk (*) denotes HSQC readouts.

Atom #	*δ*_C_	m	*δ*_H_	m	*J*_HH_ [Hz]
**2**	-				
**3**	-				
**4**	152.09	s	-		
**5**	32.33	t	2.322	dd	3.8, 18.2
			2.148	dddd	0.8, 0.8, 8.8, 18.2
**6**	76.45	d	4.008	dddd	3.8, 4.9, 7.7, 8.8
**7**	34.58	t	1.58*	m	-
			1.50*	m	-
**8**	25.21	t	1.49*	m	-
			1.39*	m	-
**9**	32.38	t	1.31*	m	-
**10**	23.28	t	1.32*	m	-
**11**	14.35	q	0.889	t	7.1
**1’**	37.57	t	2.229	m	-
**2’**	26.30	t	1.55*	m	-
**3’**	32.11	t	1.31*	m	-
**4’**	23.23	t	1.32*	m	-
**5’**	14.35	q	0.894	t	7.1
**1”**	167.17	s	-		
**2”**	37.54	t	2.753	td	6.1, 16.0
			2.717	td	6.2; 16.0
**3”**	58.84	t	3.755	ddd	5.8, 6.1, 6.2
**3”-OH**	-		2.816	t	5.8

**Fig 2:**
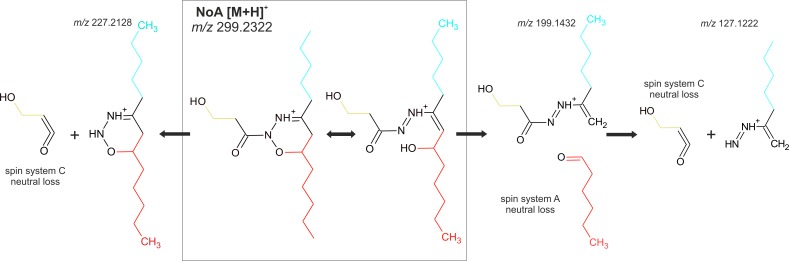
MS fragments of nocuolin A demonstrating the connection of the A/B and C spin systems obtained by NMR. We suppose the formation of secondary linear resonance structure with interrupted N-O bond of the oxadiazine ring and formation of additional N-N double bond during the ionization process. For the full MS spectra see S2 Fig and for detail fragmentation see S10 Fig.

The spin system C contains an unsubstituted hydroxyl group with an exchangeable hydrogen as confirmed by HRMS fragmentation experiments in CH_3_OH and CD_3_OD (S9 Fig). The methylenes of the spin system C have HMBC contacts to quaternary carbon C-1” at 167.17 ppm, characteristic for a carboxyl group. We further confirmed the presence of the carbonyl oxygen atom in the spin system C by obtaining the loss of C_3_H_4_O_2_, corresponding to the whole spin system C, in the HRMS fragmentation experiment (Fig 2). The connection of the spin system C to the rest of the molecule is proved by the correlation observed between methylene H-2” hydrogen of the spin system C and nitrogen atom N-2.

Based on the results described above we located all atoms in the NoA molecule with the exception of a single oxygen. Since the HMBC spectrum suggested that an atom, unobservable using NMR, is positioned between the C-6 hydrogen and nitrogen N-2 of the heterocycle, and the ^13^C and ^15^N spectra did not allow any additional bond, the oxygen was assigned to the position 1 of the heterocycle. The position was then fully confirmed by HRMS fragmentation experiments (see below).

NoA exhibited low stability in HRMS/MS experiments and thus already the MS measurement with collision energy of 0 eV revealed fragmentation of the molecular ion into fragments with *m/z* 199.1432 (C_10_H_18_N_2_O_2_ + H^+^) and 127.1222 (C_7_H_14_N_2_ + H^+^) (Fig 2 and S2A Fig). The fragment 199.1432 corresponds to the loss of the spin system B (CH_3_-CH_2_-CH_2_-CH_2_-CH_2_-CH(O)-) (∆ 1.4 ppm in FTMS) including the O-1 oxygen but lacking the terminal methylene group (Fig 2 and S6 Fig). At a higher collision energy this fragment further loses the entire spin system C together with the carbonyl group, while the two nitrogen atoms remain bonded to the spin system A (Fig 2). The presence of the two nitrogen atoms in the fragment was fully confirmed by fragmentation of ^15^N isotopically substituted NoA (S2B and S9 Figs). The presence on the N-N bond was further confirmed by a loss of molecular nitrogen in the fragmentation pathway observed in the FTMS measurement (S10A Fig). The connection of the A/B and the spin system C is demonstrated by a direct loss of the whole spin system C together with the carbonyl from the NoA molecular ion. The resulting fragment at *m/z* 227.2128 (C_12_H_26_N_2_O) shows the connection of O1 to the spin system A/B and the two nitrogen atoms. The subsequent cleavage of the product ions leads to a fragment of *m/z* 85.0401, which fits well with the elemental composition C_3_H_4_N_2_O (∆ 1.1 ppm in FTMS) of the heterocycle, suggested by NMR. The presumed presence of nitrogen atoms and exchangeable hydrogens within all fragments was in perfect agreement with the fragmentation of ^15^N labelled NoA and NoA in deuterated methanol.

**Fig 3:**
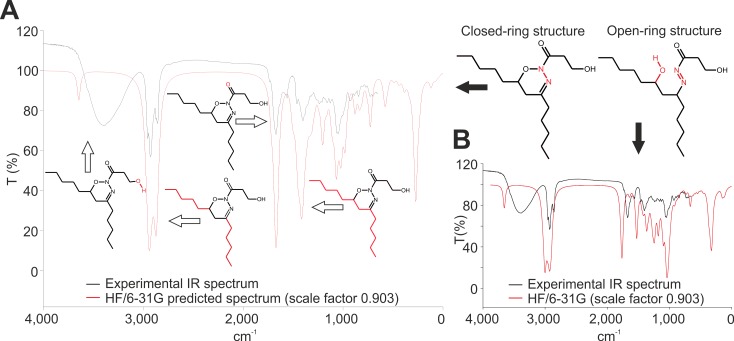
**Predicted and experimental FTIR spectra of cyclic nocuolin A form (A) and hypothetical linearized structure (B).** In the case of cyclic NoA form the theoretically predicted *ab initio* spectrum (red line) show exceptionally good overlap with the experimental (black line). Substantially different was the predicted spectrum obtained for hypothetical linear NoA form. The main vibration bands and corresponding structures are highlighted. For details see S11 and S12 Figs.

The results obtained by Fourier transform infrared spectroscopy (FTIR) confirmed the presence of a free hydroxyl group manifested as a distinctive OH stretch at 3000–4000 cm^-1^ (Fig 3). An intense double absorption peak detected at 2940 cm^-1^ resp. 2875 cm^-1^ can be explained by the stretching vibrations of both pentyl aliphatic chains, and the absorption peak at 1440 cm^-1^ corresponds to the vibration of the carbon backbones of A/B spin systems (S11 Fig). The experimental FTIR spectrum of NoA almost perfectly matched with the theoretically predicted *ab initio* spectrum (Fig 3). The fingerprint region (500–1450 cm^-1^) exhibited an exceptionally good overlap, especially when probable NoA water adducts and a NoA dimer were taken into consideration (S12 Fig). Spectra generated for alternative variants including an acyclic tautomeric form were considerably different (S11 Fig).

Based on all experimental data obtained using one- and bi-dimensional NMR techniques, HRMS, and FTIR, we concluded that the NoA molecule comprises a 1,2,3-oxadiazine heterocycle with two pentyl chains attached to carbons C-4,6 and a 3-hydroxypropanoyl chain attached to nitrogen N2. The IUPAC systematic name of NoA is 1-(4,6-dipentyl-5,6-dihydro-2H-1,2,3-oxadiazin-2-yl)-3-hydroxypropan-1-one.

### Biological activity of NoA

**Fig 4:**
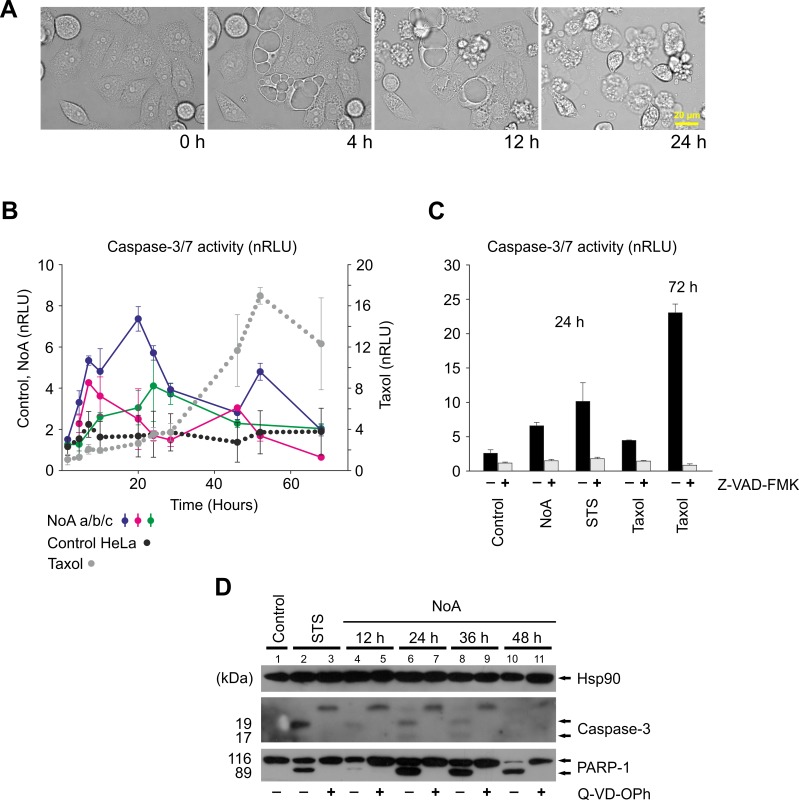
Nocuolin A triggers caspase-dependent apoptosis. (A) NoA-induced cell death morphologically resembles apoptosis (blebbing) as illustrated by the time-lapse microscopy images obtained with HeLa cells exposed to 6.7 μM NoA over 24 hours. (B, C, D) The cell death is caspase-dependent. (B) The protease activity of caspase-3/7 for the DEVD sequence was measured as a luminescence signal at various time points. The relative luminescence units were normalised per cell (nRLU). NoA (a/b/c) at 6.7 μM stands for three independent experiments. (C) The enhanced activity of caspase-3/7 (nRLU) was reduced when the cells treated with NoA and positive controls, were pre-treated with caspase inhibitor Z-VAD-FMK (10 μM). (D) NoA treatment (6.7 μM) resulted in weak enhancement of activated caspase-3 fragments (19/17 kDa) and a consistent increase in PARP-1 cleavage. These apoptosis markers were completely absent when the caspase inhibitor Q-VD-OPh (10 μM) was added prior to NoA (lines 5,7,9,11). As a positive control, taxol (B) and staurosporine (STS) (C, D) were used both at concentration 1 μM and for indicated time.

In the pilot experiments, where HeLa cells were treated with NoA, we observed extensive cytoplasmic vacuolisation beginning one hour after treatment, followed by rounding and subsequent membrane blebbing, as assessed using time-lapse microscopy (Fig 4A). Because the morphological features induced by NoA in HeLa cells strongly pointed to apoptosis, we focused on an involvement of the caspase cascade. HeLa cells treated with NoA (6.7 μM) exhibited a consistent increase in caspase-3/7 activity measured using luminescence assay in three independent time-course experiments (Fig 4B). Although the onset of caspase activation in cells treated with different batches of NoA varied among the experiments substantial proteolytical activity was observed over time in all cases (Fig 4B). The highest reading for NoA was lower in comparison to cells treated by apoptosis inducers used as positive controls (taxol and staurosporine), but it was still on average 2.5-fold higher than that in the control (Fig 4C). The subsequent decrease of the caspase-3/7 activity can be attributed to the overall degradation of the cells as also demonstrated by attenuation of ATP levels in this range (S13B Fig). The use of pan-caspase inhibitor Z-VAD-FMK consistently reduced the caspase luminescence outread for both NoA (3 μM) and positive controls (Fig 4C). As a direct evidence of active caspases, we detected the 17-kDa and 19-kDa fragments of caspase-3 at 12, 24 and 36 h after NoA treatment (Fig 4D). We did not detect any cleaved forms of caspase-7 (data not shown), and thus we conclude that caspase-3 is the main executor of NoA-mediated apoptosis. Because both methods, immunodetection and luminescence, clearly demonstrated elevated, although still rather weak caspase activity, we further investigated the processing of the important downstream caspase-substrate, Poly-(ADP-ribose)-polymerase 1 (PARP-1) (Fig 4D). Consistent with caspase-activation the accumulation of the specific 89-kDa PARP-1 fragment at 12, 24, 36 and 48 h after the addition of NoA (6.7 μM) to HeLa cells was observed. Moreover, pre-treatment with the pan-caspase inhibitor Q-VD-OPh prior to NoA addition blocked the promotion of cleaved forms of both, caspase-3 (17/19 kDa) and PARP-1 (89 kDa), confirming the caspase dependence of NoA-induced cell death (Fig 4D).

Cells from the same batch as that used for immunoblotting were analysed with flow cytometry for sub-G1 DNA content (S13A Fig). In the first 12 h of treatment with 6.7 μM NoA, the sub-G1 population was not elevated (~2.5%). The increase to 12–14% was observed at 24, 36 and 48 h; similarly as for staurosporine at 4 h. In all cases, the sub-G1 increase was blocked by pre-treatment with Q-VD-OPh, analogously to the blockage of caspase-3 and PARP-1 cleavage described earlier.

Apoptosis, in contrast to necrosis, usually requires ATP during the initiation phase of cell death. Interestingly, we observed an early ATP-level drop of NoA treated cells at 2.5–6.7 μM concentrations (S13B Fig), together with extensive vacuolisation (Fig 4A). After that the relative ATP level was stable during the first 24 h of the experiment followed by a slow decrease over time (S13B Fig

Finally, we evaluated the anti-proliferative potency of NoA against a panel of nine cancer cell lines (Table 2). The observed IC_50_ values ranged from 0.72 μM to 4.50 μM, identifying NoA as a potential anti-cancer drug-candidate. The highest effectivity was recorded in glioma U251 cells, JIMT1 and SKBR3 breast cell lines and ovarian OVCAR5 cells. Of note, increased sensitivity was actually found in the cancer lines with mutated or deleted p53 status. In particular, this was most pronounced in the two glioma cell lines tested. The IC_50_ of NoA was consistently lower in p53 mutated U251 cells (0.72 ± 0.65 μM) than in p53 wild type U87 (3.26 ± 2.13 μM), which were the most and the least sensitive cells.

**Table 2:** IC_50_ values for nocuolin A anti-proliferative activity against nine human cancer cell lines at 72 h. Metabolic activity was assessed using colorimetric PrestoBlue assay and the cell membrane permeability was measured by fluorescence probes Hoechst 33342 and Ethidium homodimer (Methods). The most sensitive and least sensitive cell lines were the glioma cancer cell lines U251 and U87, respectively. IC_50_ values equal to or below 1 μM are highlighted in bold. The p53 status is indicated as WT (wild type) or M **(**mutated); number (codon position) is followed with the sequence of WT/mutated codon, ins3 (insertion of 3 bases). The p53 status was adopted from IACR database (http://p53.iarc.fr/CellLines.aspx) and p53 Web Site (http://p53.free.fr/index.html). Values in the last column refer to the cell doubling time (h).

	Resazurin-based	Cell permeability		
Cancer cell line	IC_50_ [μM] mean ± SD	IC_50_ [μM] mean ± SD	p53 status (WT/M)	Doubling time (h)
**A549** (lung)	2.06 ± 1.03	2.46 ± 0.26	WT	~ 22
**H460** (lung)	3.03 ± 1.36	2.45 ± 1.43	WT	~ 18
**JIMT1** (breast)	**1.00 ± 0.14**	**0.88 ± 0.09**	M (248-CGG/TGG)	~ 36
**MCF7** (breast)	1.23 ± 0.28	2.62 ± 0.70	WT	~ 24
**SKBR3** (breast)	**0.76 ± 0.61**	1.94 ± 1.04	M (175-CGC/CAC)	~ 60
**BxPC3** (pancreatic)	1.47 ± 0.79	1.16 ± 0.52	M (220-TAT/TGT)	~ 24
**U251** (glioma)	**0.72 ± 0.65**	**0.95 ± 0.41**	M (273-CGT/CAT)	~ 28
**U87** (glioma)	3.26 ± 2.13	4.50 ± 2.51	WT	~ 30
**OVCAR5** (ovarian)	1.15 ± 0.36	**0.77 ± 0.22**	M (224-GAG/ins3)	~ 48

### Predicted NoA biosynthetic gene cluster

**Fig 5:**
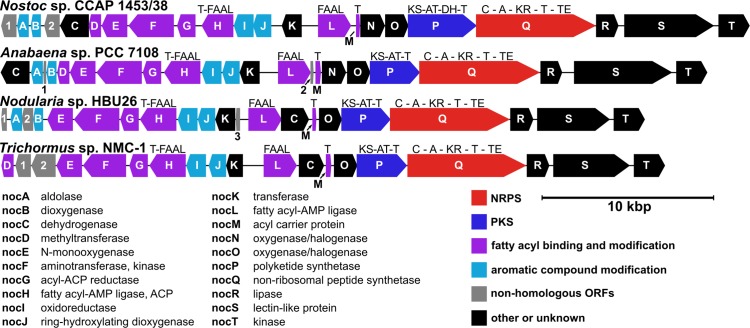
Structure of the *noc* gene cluster in four cyanobacterial strains. Gene arrangement, functional annotation of *nocA–T* genes, and domain structure of the PKS/NRPS genes. A–adenylation domain; AT–acyltransferase; C–condensation domain; DH–dehydratase domain; FAAL–fatty acyl-AMP ligase; KR–ketoreductase; KS–ketosynthetase; NRPS–non-ribosomal peptide synthetase; PKS–polyketide synthetase; T–thiolation domain (acyl or peptidyl carrier protein); TE–thioesterase. See also S1 Table.

The assembly of the *Nostoc* sp. CCAP 1453/38 and *Nodularia* sp. HBU26 DNA sequence data yielded the predicted NoA biosynthesis operon (*noc*) in the middle of a ~257 kbp and a ~266 kbp genomic scaffold, respectively. *Blastp* analysis employing the individual hypothetical Noc proteins as queries led to the identification of highly similar gene clusters in the publicly available genomes of cyanobacteria *Anabaena* sp. PCC 7108 (NZ_KB235896) and *Trichormus* sp. NMC-1 (NZ_LZQD01000000). The four gene clusters are 37,663–41,570 bp long and comprise 19–22 hypothetical protein-coding ORFs. The ORFs are in all cases presumably transcribed starting from a bidirectional promoter region, with approximately half of the genes transcribed in one direction and the remaining half in the opposite direction (Fig 5). Following HPLC-HRMS/MS measurements of *Anabaena* sp. PCC 7108 and *Nodularia* sp. HBU26 biomass extracts revealed the occurrence of a molecular ion perfectly matching that of NoA in molecular mass, fragmentation pattern and retention behaviour (S1 Fig). The genome draft of *Trichormus* sp. NMC-1 appeared only recently in the NCBI database (November 2016), thus, the production of NoA in that strain has not yet been examined.

Majority of the predicted genes in the putative NoA gene clusters of the four cyanobacterial strains (Fig 5) exhibited overall homology (S1 Table). These 20 genes were designated as *nocA*–*T*. All four *noc* clusters can be classified as hybrid PKS/NRPS clusters. They contain a single peculiar NRPS gene (*nocQ*) bearing an adenylation (A) domain with predicted specificity for aromatic amino acids (tryptophan, phenylalanine), followed by a ketoreductase (KR) domain and a thioesterase domain. Another ORF (*nocP*) was annotated as a simple PKS gene. In *Nostoc*, the predicted NocP enzyme seems to contain a dehydratase domain (DH), which is missing in the remaining three strains.

A further set of 7 ORFs (*nocD*–*H* and *nocL*–*M*) appeared to be connected to fatty acid processing, including two coding sequences of fatty acyl-AMP ligases (FAALs) and associated acyl carrier proteins (ACPs) (*nocH* and *nocL*–*M*). The adjacent genes (*nocD*–*G*) code for tailoring enzymes (methyltransferase, N-monooxygenase, aminotransferase, acyl-ACP reductase). One of them (*nocD*, predicted methyltransferase gene) is missing in the *noc* cluster of *Nodularia*, and *tblastn* analysis revealed no homologous gene in its whole genome draft.

Two consecutively encoded hypothetical proteins (NocN and NocO) with similar predicted functions appeared to be distantly related to putative acyl halogenase involved in columbamide biosynthesis (ColD). They are further (even more distantly) similar to *p*-aminobenzoate *N*-oxygenase employed in aureothin biosynthesis (AurF). Both of the *nocN* and *nocO* genes are present in the clusters of *Nostoc* and *Anabena*, while only *nocO* is retained in *Nodularia* and *Trichormus*. Therefore the exact functional annotation of these two genes needs further investigation.

Another group of predicted enzymes exhibit similarity to members of the bacterial oxidative benzene degradation pathway (ring-hydroxylating dioxygenases, aldolases, oxidoreductases; NocA–B, NocI–J). The four respective genes are present in all the three NoA-producing strains, while two of them (*nocA*–*B*) are absent in *Trichormus*, which was not tested for NoA production.

In the variable terminal part of the clusters (Fig 5, on the left), two additional putative hydrolase genes were predicted in *Nostoc*, whereas only a single truncated hydrolase was identified in *Anabaena*, a non-homologous hydrolase and a monooxygenase was found in *Nodularia*, and a methyltransferase and a cytochrome P450 oxidase in *Trichormus*. The possible tailoring activities of these enzymes require further investigation considering that only a single variant of NoA was detected and characterized in this study. Two additional truncated ORFs were predicted each in the middle of one of the *noc* clusters of *Anabaena* and *Nodularia*. The remaining proteins, encoded at the opposite end of the gene clusters, comprised a dehydrogenase (NocC), an unknown putative transferase (NocK), a lipase (NocR), a lectin-like protein (NocS), and a kinase (NocT).

Since the NocQ A-domain was predicted to specifically activate aromatic amino acids, we performed feeding experiments with isotopically substituted N^15^-tryptophan and C^13^N^15^-phenylananine (see the Methods). While tryptophan feeding led to *m/z* increase of the protonated NoA molecule by ∆1 and ∆2 resulting in equal abundance of ions 299/300/301 (S1D Fig), only a negligible change in the isotopic pattern was found in phenylalanine feeding (S1E Fig). This suggests the involvement of tryptophan in NoA synthesis either through direct activation by NocQ or through forming a precursor for activation. We suppose that the partial enrichment by the second nitrogen atom can be attributed to tryptophan degradation processes in the cell and subsequent incorporation to other intermediates entering the NoA biosynthetic pathway, e.g. as donors of an amino-group (NocF is an aminotransferase).

## Discussion

NoA establishes a novel class of natural metabolites containing a 1,2,3-oxadiazine heterocycle (N-N-O system included in a closed six-membered ring). Similar heterocyclic structures were previously reported only in synthetic compounds, mostly based on 1,3,4- or 1,2,4-oxadiazines, where the N-N-O system is interrupted by a carbon atom incorporated either between two nitrogen atoms or a nitrogen and an oxygen atom [1, 2, 4]. The actual N-N-O linkage was reported solely in five-membered rings, i.e. 1,2,3-oxadiazole derivatives [1, 5, 6]. The existence of a closed 1,2,3-oxadiazole ring was initially reported to be improbable since an open ring form is more stable, as predicted by quantum chemical modelling [29]. However, the equilibrium can be shifted towards the cyclic form by introducing adequate substituents, as demonstrated in many synthetic 1,2,3-oxadiazole derivatives [1]. The discovery of NoA brings new insights into the chemistry of heterocyclic structures containing N-N-O linkage as well as their natural occurrence. In this study we report for the first time the existence of an organic compound based on the six-membered 1,2,3-oxadiazine heterocycle, intriguingly isolated from a biological source.

From the pharmaceutical perspective, the 1,3,4-oxadiazoles in particular represent a considerable platform for drug development [2, 8, 30, 31]. Since oxadiazole structures were demonstrated to exhibit a broad range of biological activity, there is a strong interest in developing new 1,3,4-oxadiazole derivatives. To date, compounds with anti-inflammatory, antiallergic, antipsychotic, antimicrobial, antitumour, and antiviral effects were reported within this class [2]. The six-membered oxadiazines were also reported as potential lead compounds. Synthetic 1,3,4-oxadiazine derivatives manifested high efficiencies against cancer cells in nanomolar range [4]. Cancer cells are frequently escaping cell death, yet drugs inducing apoptosis are still a major pillar of current cancer therapies. A majority of lead compounds were inspired by natural secondary metabolites. Our data clearly demonstrate that the cell death induced by NoA is consistent with apoptosis (blebbing, caspase activation and PARP-1 cleavage). Loss of p53 function is known to be responsible for drug-resistance in more than half of human cancers [32]. Apparently NoA induced cell death is independent of p53 function since we have observed full activation of caspase-3 and PARP-1 cleavage in HeLa cells lacking functional p53 and Rb protein [33]. On top of that p53 mutant cell lines exhibited even higher sensitivity to NoA best detectable in U251 glioma and JIMT1 breast cancer cells. The ability to induce apoptosis in p53 mutated cell lines makes NoA an interesting lead.

Our study demonstrates that cyanobacteria are capable of synthetizing bioactive compounds based on the oxadiazine heterocycles, which are generally known to have promising pharmaceutical properties. This knowledge opens new possibilities for a broader natural oxadiazine screening. The cyanobacterial origin of NoA is evidenced through the production of the compound by one axenic (*Anabaena* sp. PCC 7108) and two uni-cyanobacterial strains (*Nostoc* sp. CCAP 1453/38 and *Nodularia* sp. HBU26).

We identified peculiar, homologous biosynthetic gene clusters in the genomes of all the three NoA-producing strains. The draft genomes of *Nostoc* CCAP 1453/38 and *Nodularia* HBU26 were sequenced in this study, while the complete genome of *Anabaena* sp. PCC 7108 is publicly available. Another highly similar cluster (although lacking two tailoring genes) was mined from the genome draft of *Trichormus* sp. NMC-1, which has recently appeared in the NCBI database [34]. The production of NoA in this strain was not studied. In favour of designation of *noc* as the NoA biosynthesis operon, this (or similar) cluster has not been identified in any other sequenced genome of any other organism. The proposed *noc* cluster represents a unique combination of PKS/NRPS [16,17] enzymes activating fatty acid residues [35], an aromatic ring degradation cascade [36,37], and associated tailoring enzymes. Although the proposed operon is largely novel and the biosynthetic pathway is far from explained, results presented above support the hypothesis that the *noc* operon is responsible for NoA synthesis. The evidence is further strengthened by experiments using ^15^N isotopically substituted tryptophan, which revealed incorporation of the tryptophan nitrogen into NoA molecule. This is in agreement with the predicted NRPS A-domain specificity for an aromatic amino-acid. The presence of two encoded FAAL enzymes typical for lipopeptide synthetases [38] could be further linked with the structure of NoA, which contains two aliphatic side chains. An almost complete set of enzymes matching the known oxidative benzene degradation pathway in bacteria [36,37] may explain the absence of an aromatic ring in the structure, which is normally expected as long as tryptophan is utilized as a substrate. Extensive genetic manipulation and biochemical assays are required to gain further mechanistic insight into the individual biosynthetic steps and to provide conclusive evidence concerning the role of the predicted *noc* gene cluster in the production of NoA.

## Supporting information

S1 Fig HPLC-MS/MS chromatograms of NoA-producing strains. (A) BPC (base peak chromatogram) of *Nostoc* sp. CCAP 1453/38 extract, EIC (extracted ion chromatogram) of NoA is highlighted in red, HRMS/MS spectrum of NoA (upper-right corner). (B) BPC of *Anabaena* sp. PCC 7108 extract, EIC of NoA is highlighted in red, HRMS/MS spectrum of NoA (upper-right corner). The peak marked with an asterisk corresponds to an ion of m/z 619.4379 with elemental composition different from NoA. (C) BPC of *Nodularia* sp. HBU26 extract, EIC of NoA is highlighted in red, HRMS/MS spectrum of NoA (upper-right corner). (D) BPC of *Nostoc* sp. CCAP 1453/38 extract after ^15^N-tryptophan feeding, EIC of NoA is highlighted in red, HRMS/MS spectrum of NoA (upper-right corner) is showing the corresponding mass shift. (E) BPC of *Nostoc* sp. CCAP 1453/38 extract after ^13^C^15^N-phenylalanine feeding, EIC of NoA is highlighted in red, HRMS/MS spectrum of NoA (upper-right corner).
(TIF)

S2 Fig HRMS and HRMS/MS spectra of NoA. (A) HRMS spectrum of NoA. NoA exhibited low stability in MS/MS experiments and thus even the MS measurement using collision energy of 0 eV revealed minor cleavage. (B) HRMS/MS spectrum of NoA at 35 eV. (C) HRMS spectrum of ^15^N isotopically substituted NoA. The relative increase of mass in the molecular ion, sodium and potassium adduct by 2 Da and the relative increase of mass in the NoA dimer by 4 Da proves the presence of two nitrogen atoms in the NoA molecule. (D) HRMS/MS spectrum of ^15^N isotopically substituted NoA.
(TIF)

S3 Fig ^13^C NMR spectrum of NoA. (TIF)

S4 Fig ^1^H-^13^C HSQC spectrum of NoA. (TIF)

S5 Fig ^1^H NMR spectrum of NoA. (TIF)

S6 Fig NMR COSY spectrum of NoA. (TIF)

S7 Fig ^1^H-^13^C HMBC spectrum of NoA. (TIF)

S8 Fig ^1^H-^15^N NMR spectrum of ^15^N-labled NoA. (TIF)

S9 Fig Fragmentation of NoA in deuterated methanol (CD_3_OD). (A) NoA MS spectrum in CD_3_OD. The mass shift by 1 Da observed in NoA sodium adduct demonstrates the presence of a single exchangeable proton in the molecule. (B) MS/MS spectrum of NoA in CD_3_OD. (C) Interpretation of main fragments in CH_3_OH and CD_3_OD.
(TIF)

S10 Fig Fragmentation of NoA obtained in HRMS and FTMS measurements. Fragmentation experiments suggested two possible fragmentation pathways of the NoA molecular ion. The first pathway occurs both in qTOF and FTMS instruments (fragments 1–10); the second pathway (11–21) requires intramolecular rearrangement and occurs mainly in FTMS. (†Data from HRMS (qTOF, Bruker Impact), ‡Data from FTMS (Bruker, Solarix).
(TIF)

S11 Fig Computationally predicted and experimental FTIR spectra of possible NoA structural variants. (A) Interpretation of major infrared absorption peaks of NoA: Absorption in the region 3000–4000 cm^-1^ corresponds to the OH stretch; the strong double absorption peak at 2940 cm^-1^ resp. 2875 cm^-1^ can be explained by the vibration of both pentyl aliphatic chains, just as the absorption around 1440 cm^-1^ by the vibration of the whole carbon structure. (B) Prediction of the infrared spectrum of other potential NoA structures. None of the other hypothetical structures exhibited good agreement with the experimental data.
(TIF)

S12 Fig Cumulative IR spectrum of different NoA adducts including NoA dimer. Cumulative predicted spectrum was modelled for a mixture of pure NoA (A), NoA dimer and different water (B) and methanol adducts (C) at an equal ratio. The fingerprint region of obtained experimental FTIR fits our predicted cumulative spectrum; the sharp peak at 1560 cm^-1^ corresponds to water absorption just as the broad peak between 3,000 and 4,000 cm^-1^, which overlays unique absorption bands of NoA in this region. (B) Predicted IR spectra of different NoA water adducts. (C) Predicted IR spectra of different NoA methanol adducts.
(TIF)

S13 Fig Cell death markers: sub-G1 population and ATP content. (A) PI staining for sub-G1 determination by FACS. Columns A-K in the graph in the upper left corner show percentage of the sub-G1 population in each sample, panels A-K depict the corresponding FACS plots. The increase of the sub-G1 population observed in HeLa cells exposed to 6.7 μM NoA between 24 and 48 h was similar to that detected in cells treated with 1 μM staurosporine (STS) for 4 h. In untreated control cells the sub-G1 population was below 1%. Pre-treatment of cells with the caspase inhibitor Q-VD-OPh (10 μM) prior to exposure to STS or NoA prevented the increase of the subG1 population (columns c/e/g/i/k). (B) Time-dependent changes in cellular ATP levels at different NoA concentrations. Interestingly, there was an ATP drop in early time points for NoA at concentrations of 2.5 and 6.7 μM. For NoA at a concentration of 6.7 μM this was followed by a further slow, but stable decrease in the relative ATP content from the 10 h time point on. In contrast, ATP level in cells treated with 2.5 μM NoA returned to the original level and was maintained for the next 50 h. Cells treated with a lower NoA concentration (0.34 μM) exhibited only a slightly diminished ATP content compared to the control.
(TIF)

S1 Table Deduced functions of the open reading frames in the *noc* gene clusters from *Nostoc* sp. CCAP 1453/38, *Anabaena* sp. PCC 7108, *Nodularia* sp. HBU 26, and *Trichormus* sp. NMC-1. ^a^Pairwise amino acid sequence identity between CCAP 1453/38 and each of the other three strains (rounded to integer values). ^b^*Nostoc* sp. CCAP 1453/38 was used as the query organism, and hypothetical proteins lacking annotation were excluded from the best-scoring hits. ACP–acyl carrier protein; FAAL–fatty acyl-AMP ligase; PKS–polyketide synthase; NRPS–non-ribosomal peptide synthase.
(DOCX)
