# Supplementary figures and images for: The cyanobacterial metabolite nocuolin a is a natural oxadiazine that triggers apoptosis in human cancer cells
Source: PLoS One. 2017 Mar 2;12(3):e0172850. doi: 10.1371/journal.pone.0172850 (PMC5333925; doi:10.1371/journal.pone.0172850)

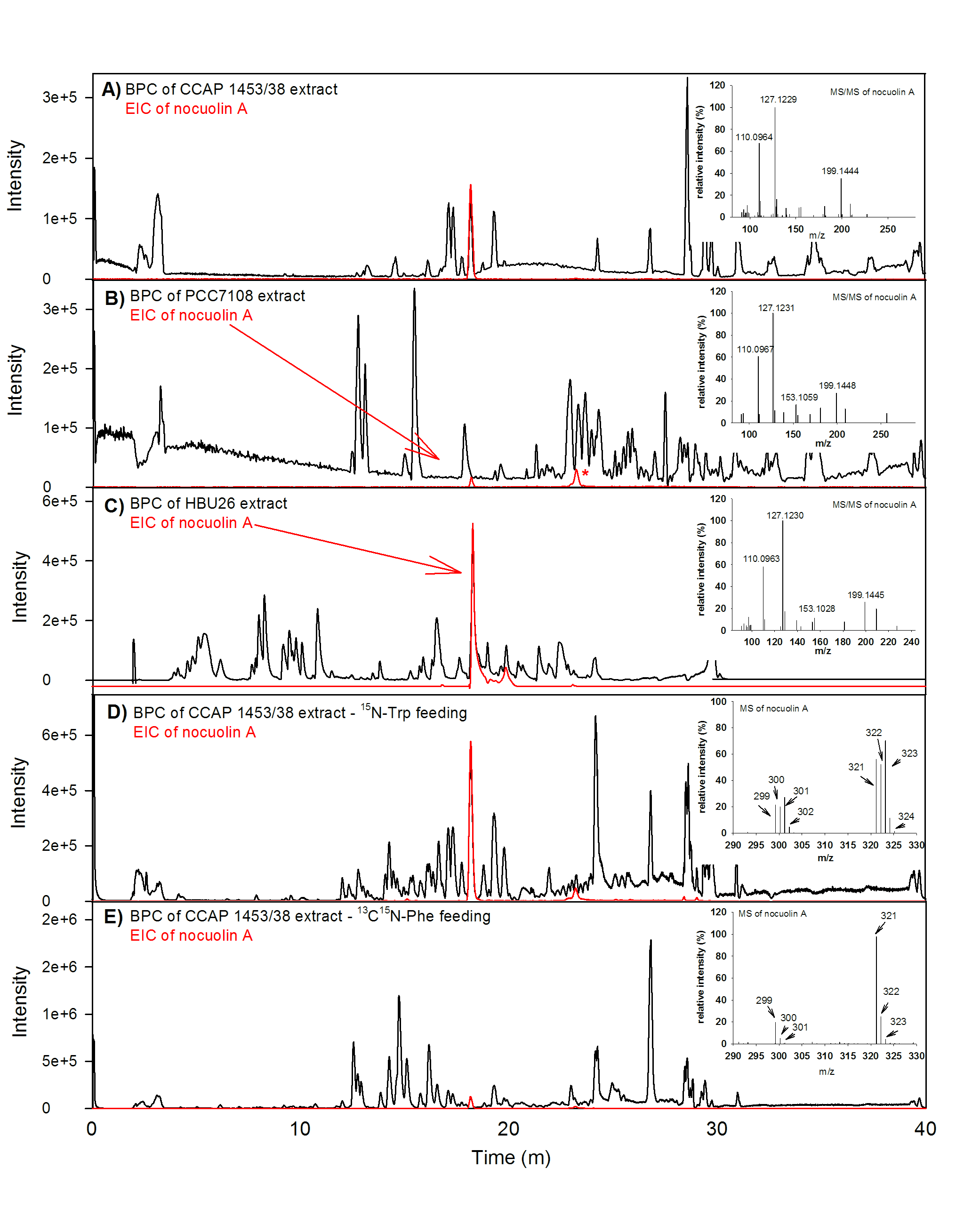

Supplement: S1 Fig — (A) BPC (base peak chromatogram) of Nostoc sp. CCAP 1453/38 extract, EIC (extracted ion chromatogram) of NoA is highlighted in red, HRMS/MS spectrum of NoA (upper-right corner). (B) BPC of Anabaena sp. PCC 7108 extract, EIC of NoA is highlighted in red, HRMS/MS spectrum of NoA (upper-right corner). The peak marked with an asterisk corresponds to an ion of m/z 619.4379 with elemental composition different from NoA. (C) BPC of Nodularia sp. HBU26 extract, EIC of NoA is highlighted in red, HRMS/MS spectrum of NoA (upper-right corner). (D) BPC of Nostoc sp. CCAP 1453/38 extract after 15N-tryptophan feeding, EIC of NoA is highlighted in red, HRMS/MS spectrum of NoA (upper-right corner) is showing the corresponding mass shift. (E) BPC of Nostoc sp. CCAP 1453/38 extract after 13C15N-phenylalanine feeding, EIC of NoA is highlighted in red, HRMS/MS spectrum of NoA (upper-right corner). (TIF) [file pone.0172850.s001.tif]

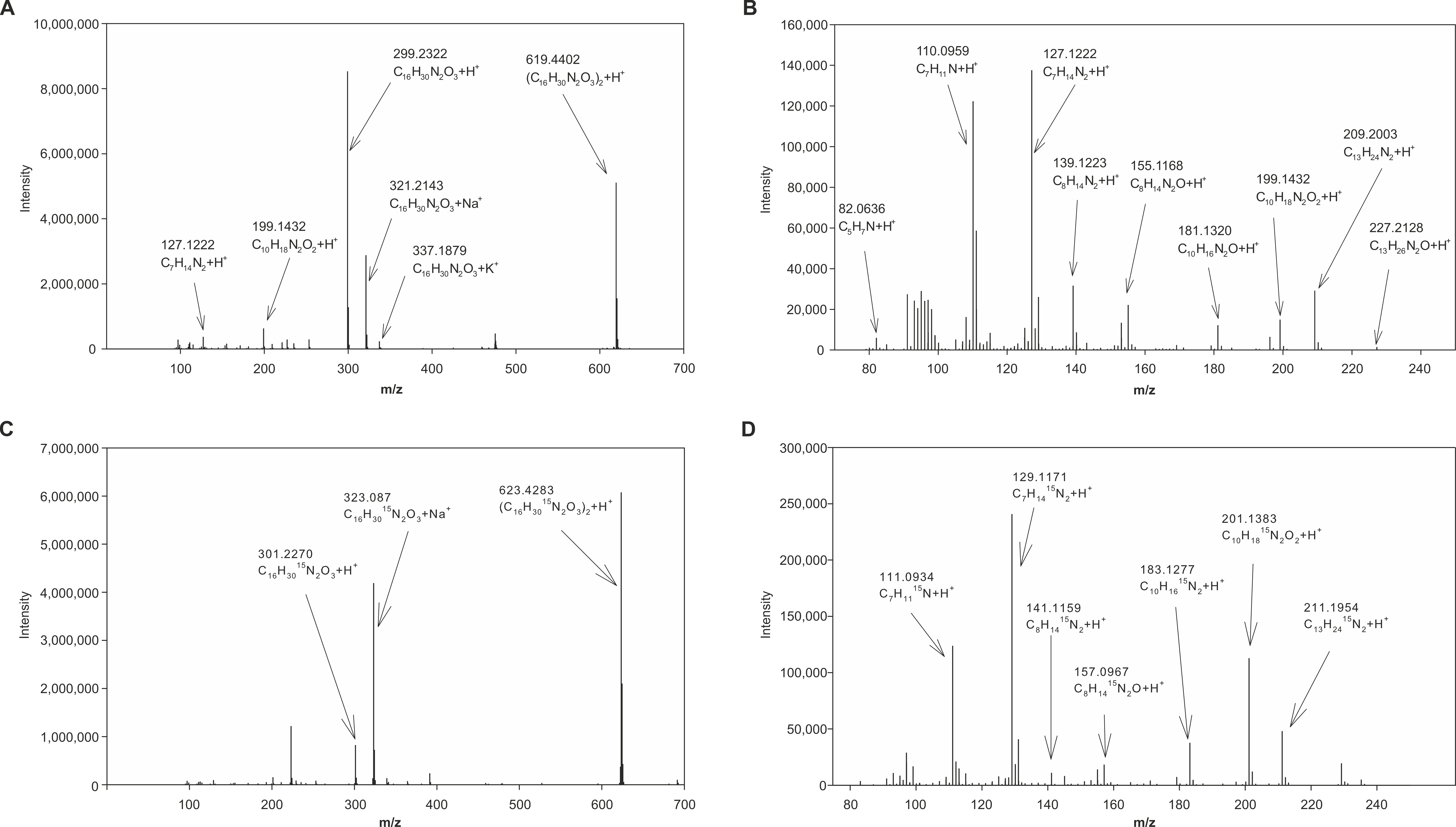

Supplement: S2 Fig — (A) HRMS spectrum of NoA. NoA exhibited low stability in MS/MS experiments and thus even the MS measurement using collision energy of 0 eV revealed minor cleavage. (B) HRMS/MS spectrum of NoA at 35 eV. (C) HRMS spectrum of 15N isotopically substituted NoA. The relative increase of mass in the molecular ion, sodium and potassium adduct by 2 Da and the relative increase of mass in the NoA dimer by 4 Da proves the presence of two nitrogen atoms in the NoA molecule. (D) HRMS/MS spectrum of 15N isotopically substituted NoA. (TIF) [file pone.0172850.s002.tif]

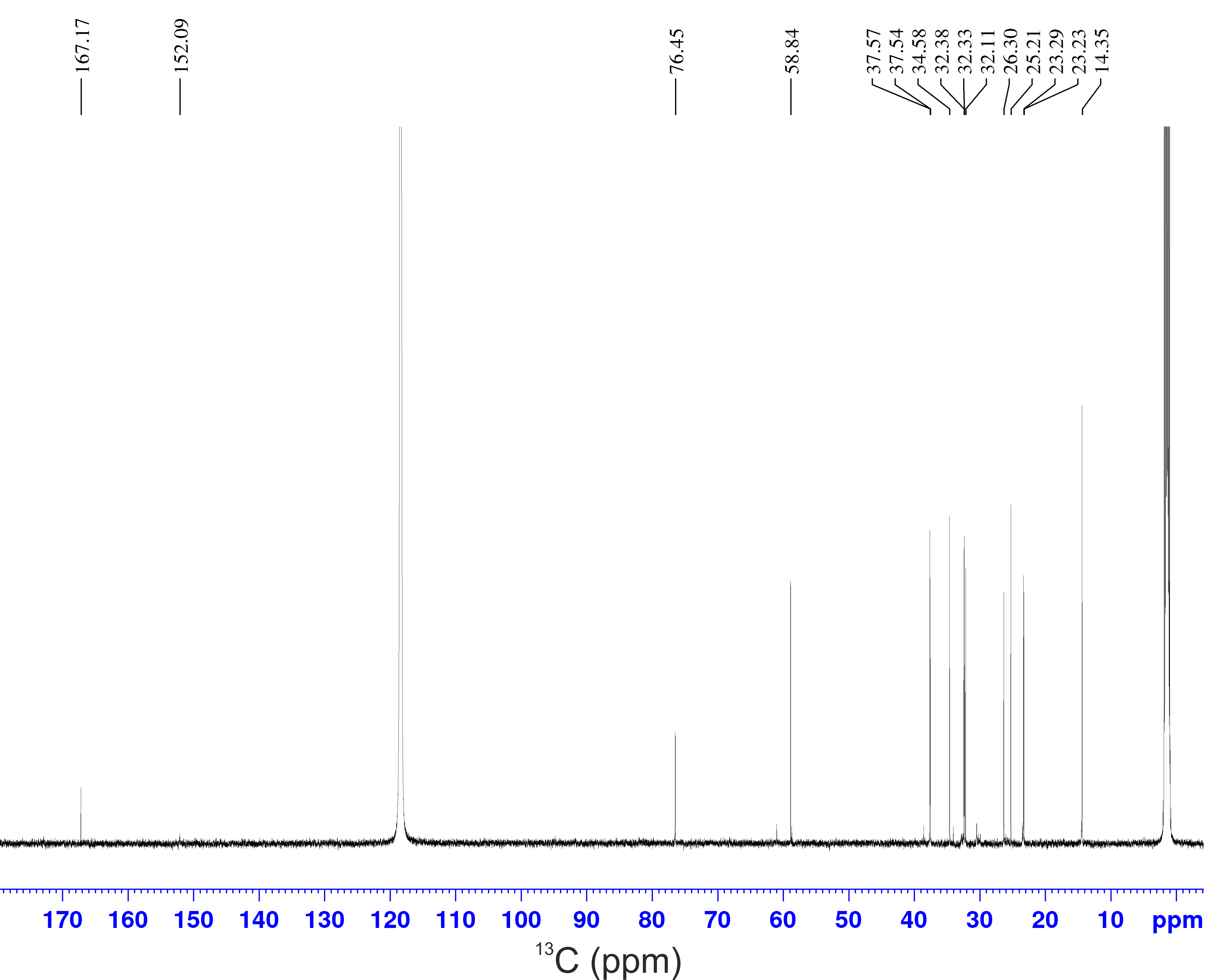

Supplement: S3 Fig — (TIF) [file pone.0172850.s003.tif]

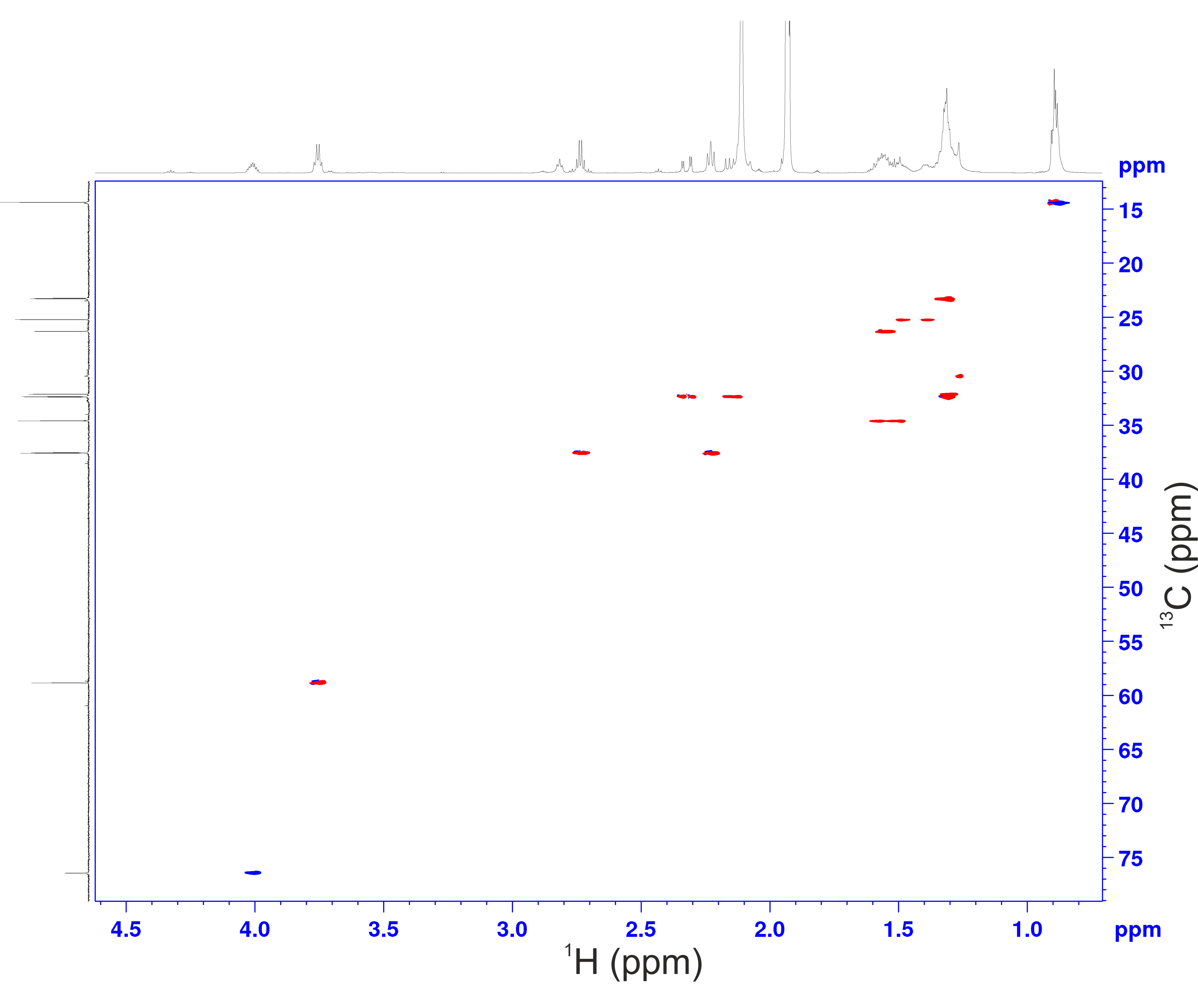

Supplement: S4 Fig — (TIF) [file pone.0172850.s004.tif]

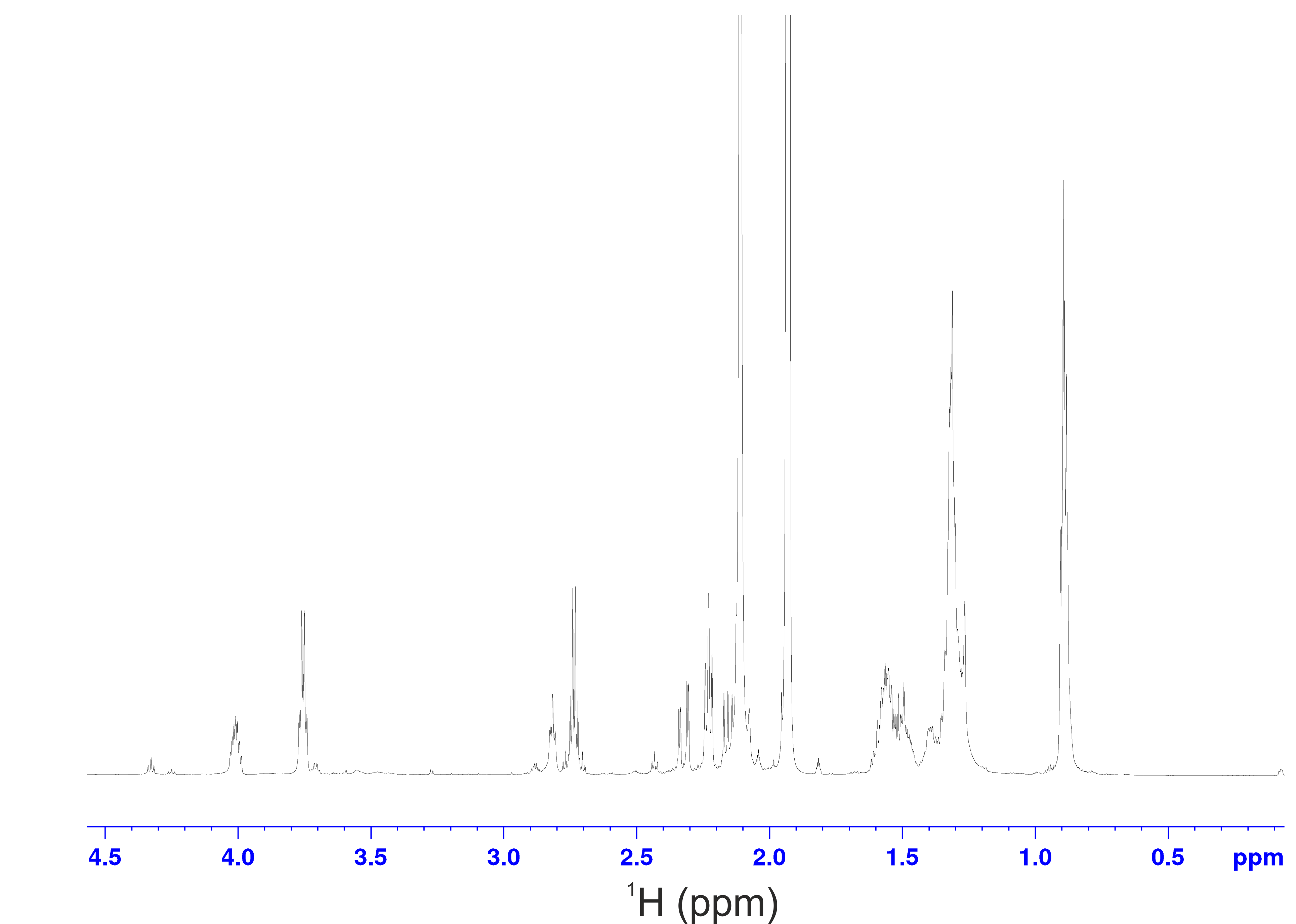

Supplement: S5 Fig — (TIF) [file pone.0172850.s005.tif]

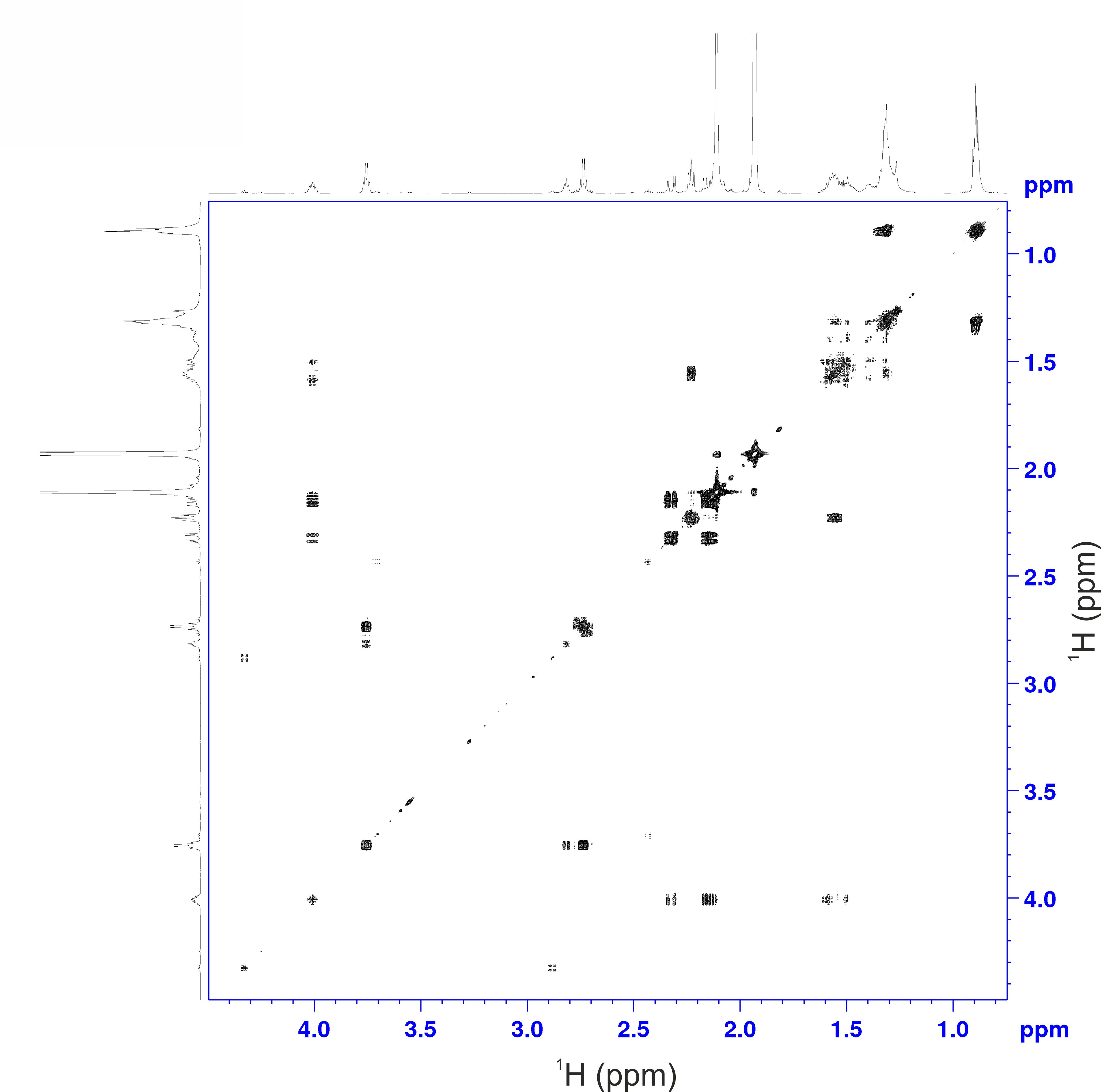

Supplement: S6 Fig — (TIF) [file pone.0172850.s006.tif]

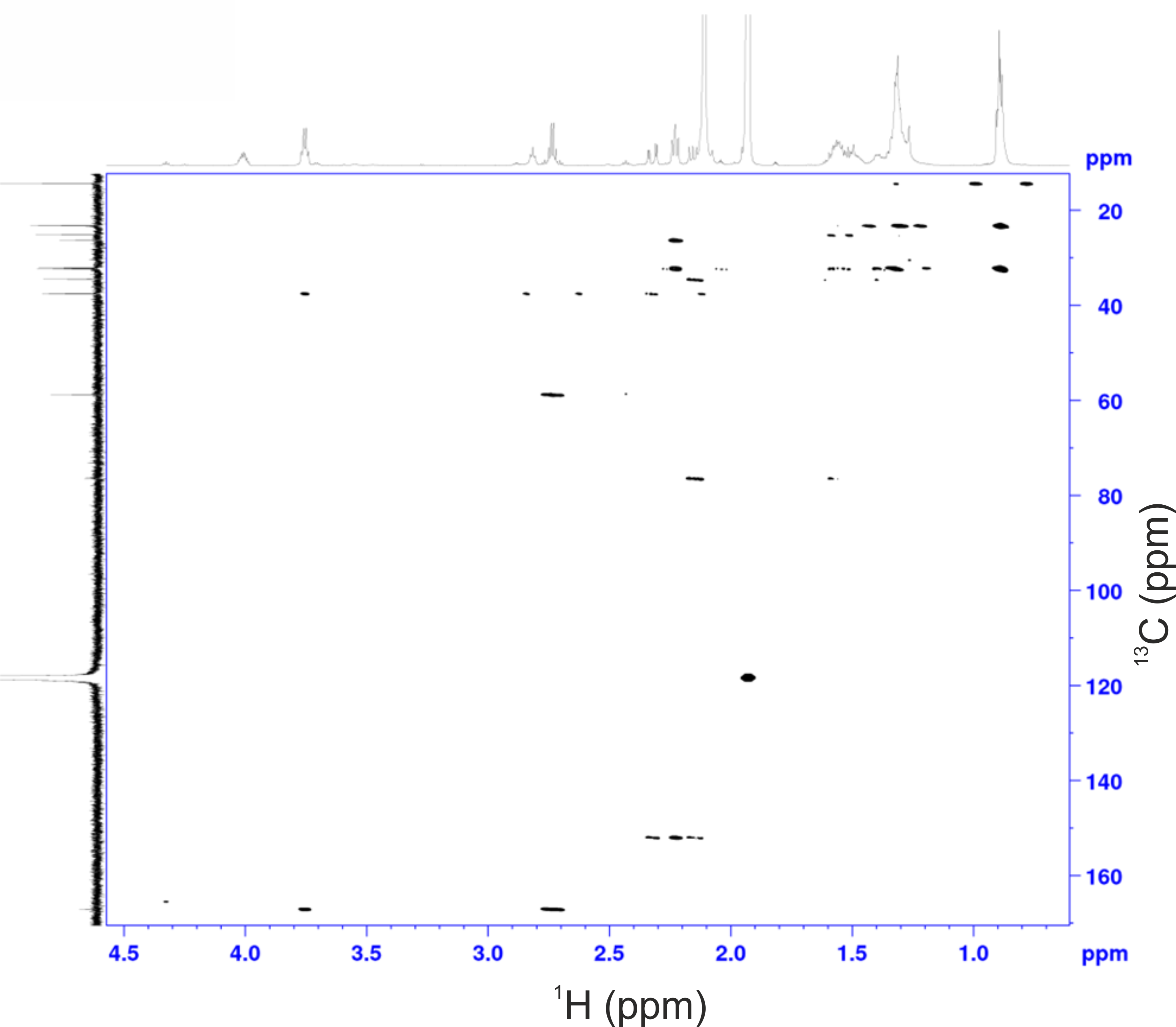

Supplement: S7 Fig — (TIF) [file pone.0172850.s007.tif]

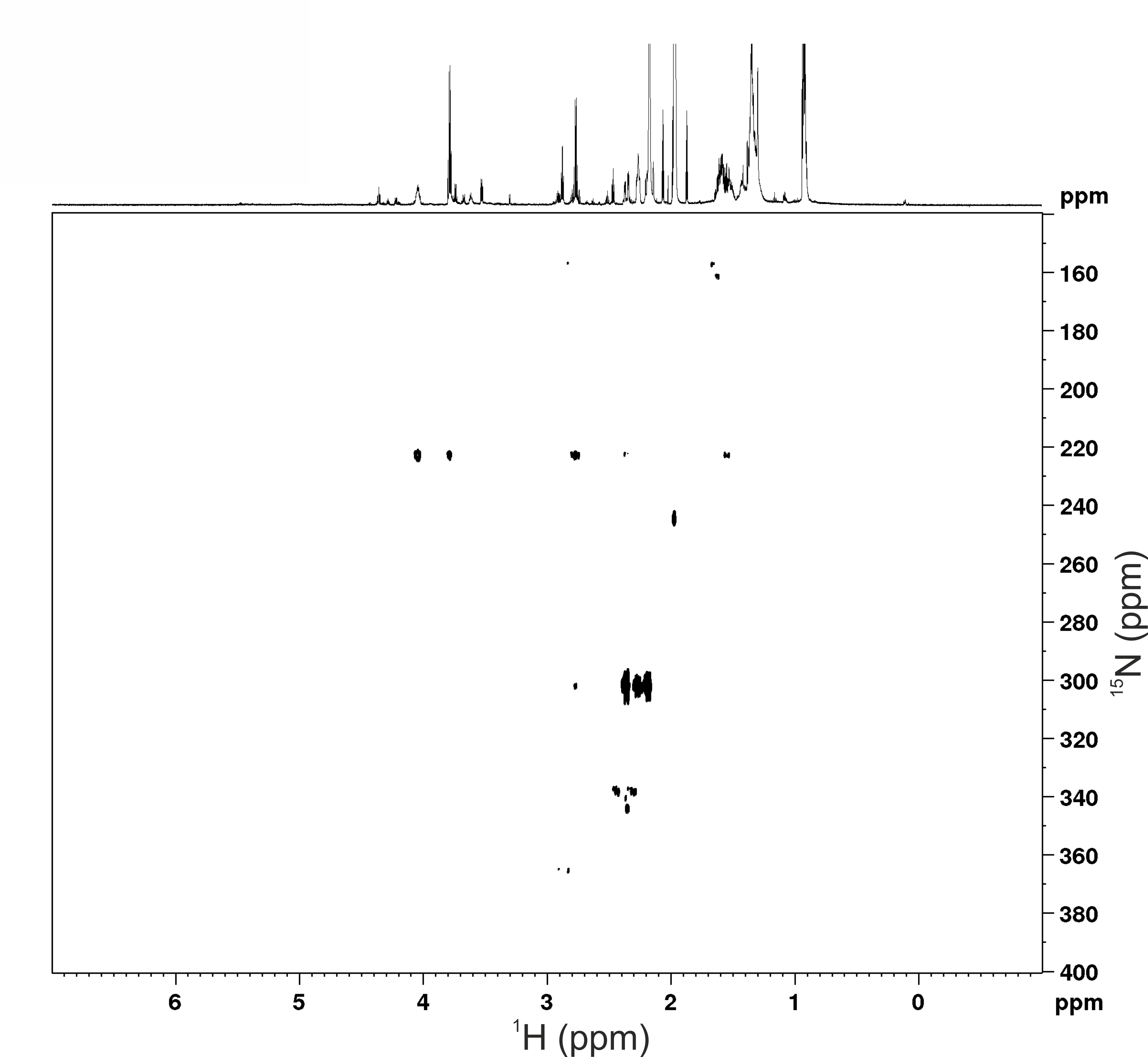

Supplement: S8 Fig — (TIF) [file pone.0172850.s008.tif]

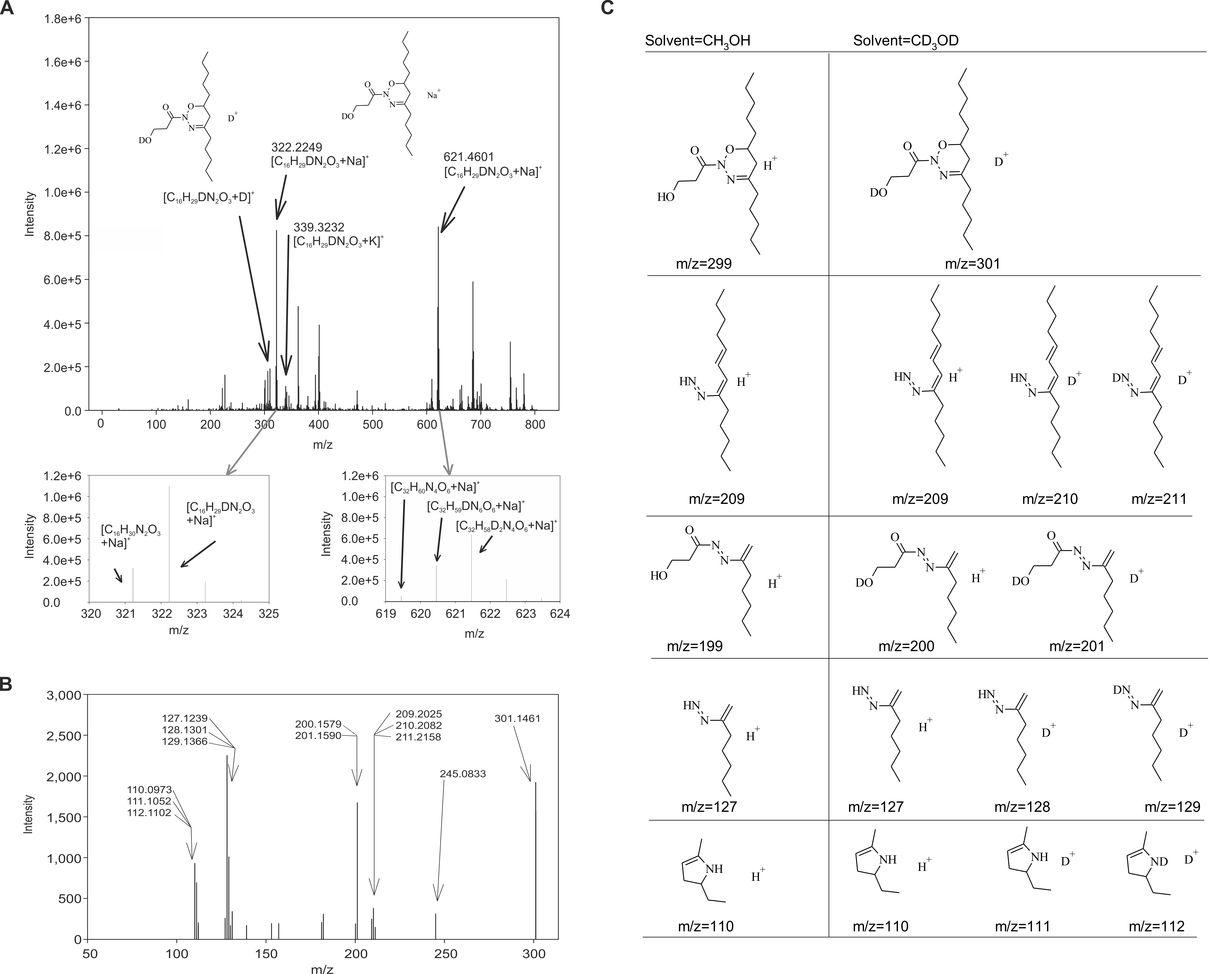

Supplement: S9 Fig — (A) NoA MS spectrum in CD3OD. The mass shift by 1 Da observed in NoA sodium adduct demonstrates the presence of a single exchangeable proton in the molecule. (B) MS/MS spectrum of NoA in CD3OD. (C) Interpretation of main fragments in CH3OH and CD3OD. (TIF) [file pone.0172850.s009.tif]

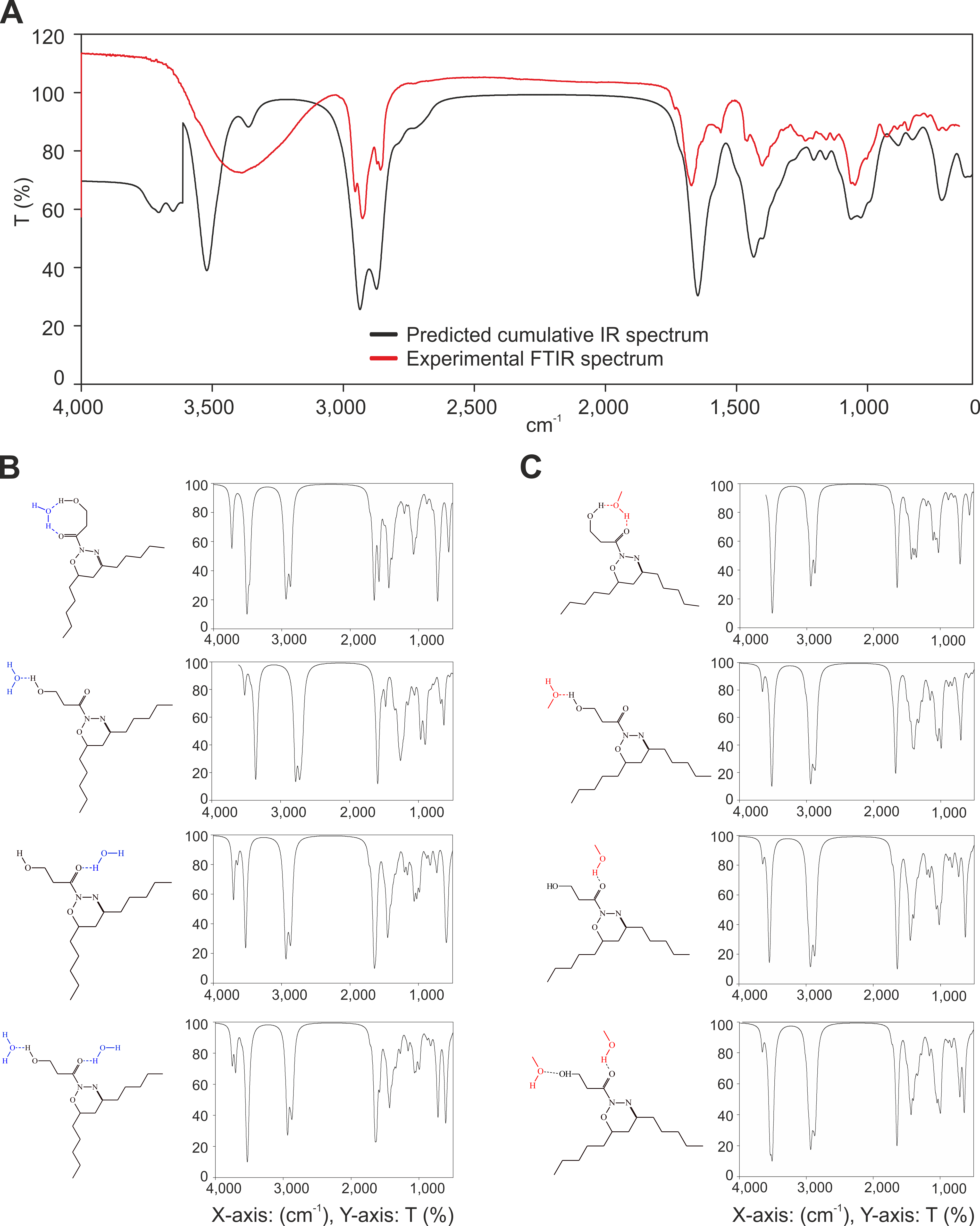

Supplement: S12 Fig — Cumulative predicted spectrum was modelled for a mixture of pure NoA (A), NoA dimer and different water (B) and methanol adducts (C) at an equal ratio. The fingerprint region of obtained experimental FTIR fits our predicted cumulative spectrum; the sharp peak at 1560 cm-1 corresponds to water absorption just as the broad peak between 3,000 and 4,000 cm-1, which overlays unique absorption bands of NoA in this region. (B) Predicted IR spectra of different NoA water adducts. (C) Predicted IR spectra of different NoA methanol adducts. (TIF) [file pone.0172850.s012.tif]

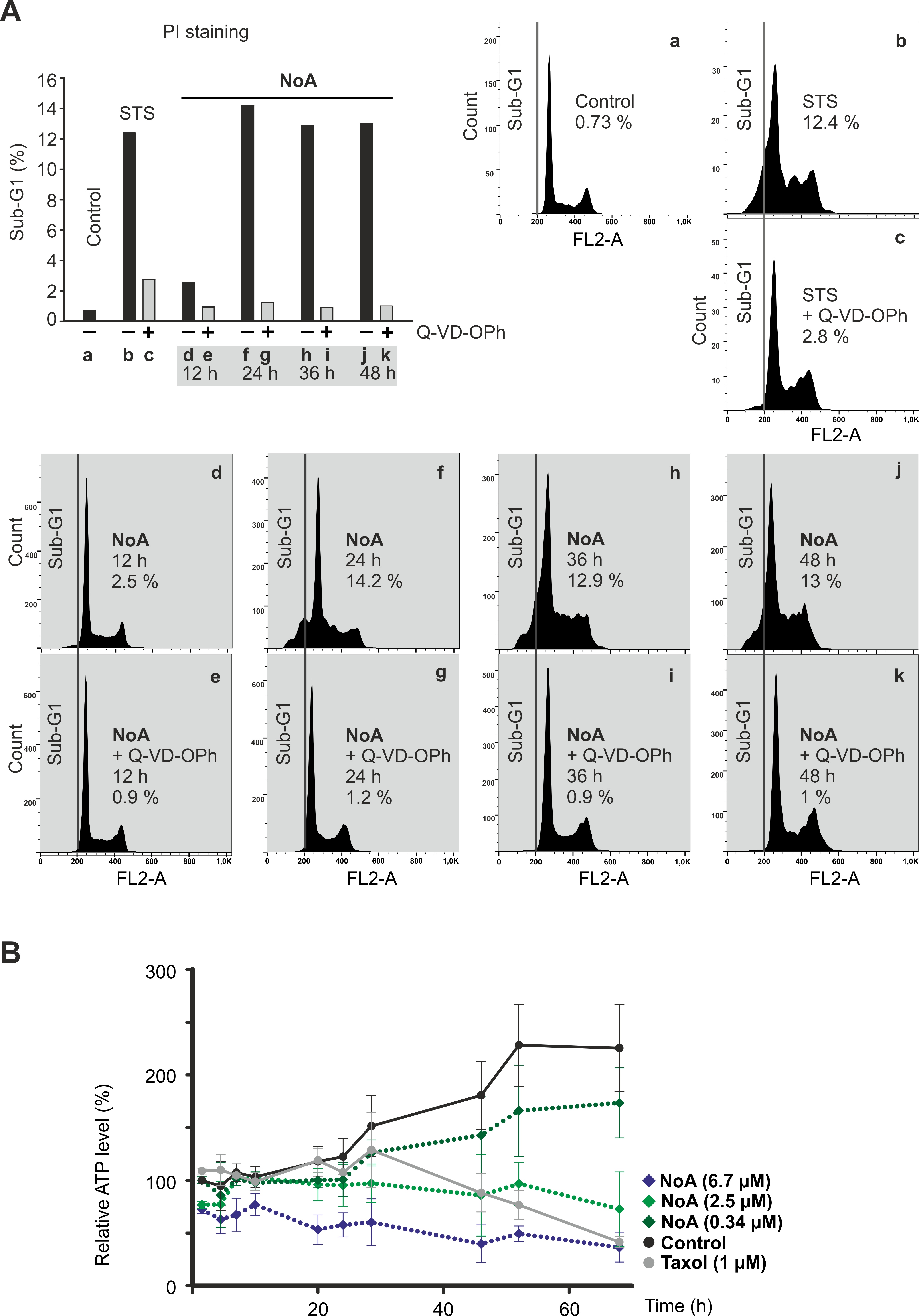

Supplement: S13 Fig — (A) PI staining for sub-G1 determination by FACS. Columns A-K in the graph in the upper left corner show percentage of the sub-G1 population in each sample, panels A-K depict the corresponding FACS plots. The increase of the sub-G1 population observed in HeLa cells exposed to 6.7 μM NoA between 24 and 48 h was similar to that detected in cells treated with 1 μM staurosporine (STS) for 4 h. In untreated control cells the sub-G1 population was below 1%. Pre-treatment of cells with the caspase inhibitor Q-VD-OPh (10 μM) prior to exposure to STS or NoA prevented the increase of the subG1 population (columns c/e/g/i/k). (B) Time-dependent changes in cellular ATP levels at different NoA concentrations. Interestingly, there was an ATP drop in early time points for NoA at concentrations of 2.5 and 6.7 μM. For NoA at a concentration of 6.7 μM this was followed by a further slow, but stable decrease in the relative ATP content from the 10 h time point on. In contrast, ATP level in cells treated with 2.5 μM NoA returned to the original level and was maintained for the next 50 h. Cells treated with a lower NoA concentration (0.34 μM) exhibited only a slightly diminished ATP content compared to the control. (TIF) [file pone.0172850.s013.tif]
