# Supplementary material for: The cyanobacterial metabolite nocuolin a is a natural oxadiazine that triggers apoptosis in human cancer cells
Source: PLoS One. 2017 Mar 2;12(3):e0172850. doi: 10.1371/journal.pone.0172850 (PMC5333925; doi:10.1371/journal.pone.0172850)
Supplement: S1 Table — aPairwise amino acid sequence identity between CCAP 1453/38 and each of the other three strains (rounded to integer values). bNostoc sp. CCAP 1453/38 was used as the query organism, and hypothetical proteins lacking annotation were excluded from the best-scoring hits. ACP–acyl carrier protein; FAAL–fatty acyl-AMP ligase; PKS–polyketide synthase; NRPS–non-ribosomal peptide synthase. (DOCX) [file pone.0172850.s014.docx]

| **Protein** | **Predicted Function** | **Length [AA]** | | | | **Pairwise Identity [%]^a^** | | | **Best-Scoring Hit (*blastp*)^b^** | | | |
| --- | --- | --- | --- | --- | --- | --- | --- | --- | --- | --- | --- | --- |
|  |  | **CCAP 1453/38** | **PCC 7108** | **HBU 26** | **NMC-1** | **PCC 7108** | **HBU26** | **NMC-1** | **Organism** | **Protein** | **Accession** | **Pairwise Identity [%]** |
| NocA | aldolase | 214 | 217 | 215 | missing | 83 | 83 | N/A | *Fischerella* sp. PCC 9431 | methylthioribulose-1-phosphate dehydratase | WP_026719419 | 71 |
| NocB | dioxygenase | 185 | 184 | 184 | missing | 78 | 78 | N/A | *Nostoc* sp. NIES-3756 | acireductone dioxygenase | WP_067763884 | 76 |
| NocC | dehydrogenase | 550 | 550 | 535 | 479 | 76 | 46 | 44 | *Cyanothece* sp. PCC 7822 | acyl-CoA dehydrogenase domain-containing protein | WP_013334490 | 62 |
| NocD | methyltransferase | 231 | 225 | missing | 225 | 74 | N/A | 67 | *Pseudomonas fluorescens* | isoprenylcysteine carboxyl methyltransferase | WP_016986888 | 34 |
| NocE | N-monooxygenase | 486 | 486 | 486 | 486 | 85 | 86 | 87 | *Gloeobacter violaceus* | dimethylaniline monoxygenase | WP_011141962 | 49 |
| NocF | aminotrasferase+kinase | 872 | 872 | 872 | 827 | 89 | 90 | 90 | *Tolypothrix campylonemoides* | aminotransferase | WP_041033409 | 88 |
| NocG | acyl-ACP reductase | 347 | 347 | 347 | 347 | 86 | 88 | 90 | *Tolypothrix campylonemoides* | 3-oxoacyl-ACP synthase | WP_041033411 | 87 |
| NocH | FAAL | 696 | 697 | 697 | 696 | 82 | 81 | 83 | *Tolypothrix campylonemoides* | AMP-dependent synthetase | WP_052490175 | 82 |
| NocI | oxidoreductase | 373 | 373 | 373 | 373 | 77 | 85 | 77 | *Anabaena* sp. WA93 | oxidoreductase | OBQ22661 | 58 |
| NocJ | ring-hydroxylating dioxygenase | 344 | 327 | 327 | 327 | 87 | 91 | 92 | *Trichodesmium erythraeum* | (2Fe-2S)-binding protein | WP_011610415 | 38 |
| NocK | transferase | 376 | 308 | 377 | 314 | 78 | 81 | 77 | *Candidatus Rubidus massiliensis* | S-adenosylmethionine: diacylglycerol 3-amino-3-carboxypropyl transferase | CDZ81690 | 32 |
| NocL | FAAL | 634 | 628 | 639 | 635 | 82 | 82 | 80 | *Scytonema tolypothrichoides* | AMP-dependent synthetase | WP_048867317 | 65 |
| NocM | ACP | 65 | 94 | 65 | 94 | 77 | 77 | 74 | *Moorea producens* | phosphopantetheine-binding protein | WP_070396823 | 73 |
| NocN | oxygenase/halogenase | 468 | 459 | missing | missing | 82 | N/A | N/A | *Moorea bouillonii* PNG5-198 | ColD (putative halogenase) | AKQ09581 | 53 |
| NocO | oxygenase/halogenase | 442 | 442 | 434 | 442 | 86 | 76 | 80 | *Moorea bouillonii* PNG5-198 | ColD (putative halogenase) | AKQ09581 | 44 |
| NocP | PKS | 1330 | 965 | 949 | 969 | 57 | 58 | 59 | *Moorea producens* | beta-ketoacyl synthase | WP_070394217 | 58 |
| NocQ | NRPS | 2324 | 2338 | 2322 | 2303 | 81 | 82 | 82 | *Lyngbya majuscula* | HctE (NRPS) | AAY42397 | 46 |
| NocR | lipase | 426 | 411 | 438 | 427 | 66 | 75 | 75 | *Paenibacillus* sp. 32O-W | lipase | WP_062491063 | 34 |
| NocS | lectin-like protein | 1070 | 1903 | 1406 | 1479 | 31 | 42 | 54 | *Microcystis aeruginosa* | 5'-nucleotidase | WP_044033948 | 50 |
| NocT | kinase | 597 | 594 | 596 | 596 | 74 | 77 | 78 | *Crinalium epipsammum* | ABC-1 domain-containing protein | WP_015202593 | 47 |
| ORF1 | hydrolase | 287 | missing | missing | missing | N/A | N/A | N/A | *Halogeometricum rufum* | Pimeloyl-ACP methyl ester carboxylesterase | SFR75953 | 46 |
| ORF2 | hydrolase | 289 | missing | missing | missing | N/A | N/A | N/A | *Ktedonobacter racemifer* | alpha/beta hydrolase fold protein | WP_007908759 | 49 |
| ORF1 | hydrolase fragment | missing | 44 | missing | missing | N/A | N/A | N/A | *Calothrix* sp. PCC 7507 | haloacid dehalogenase | WP_015127684 | 79 |
| ORF2 | FAAL fragment | missing | 48 | missing | missing | N/A | N/A | N/A | *Nostoc* sp. PCC 7120 | AMP-dependent ligase | WP_010996834 | 50 |
| ORF1 | monooxygenase | missing | missing | 99 | missing | N/A | N/A | N/A | *Calothrix* sp. PCC 7507 | antibiotic biosynthesis monooxygenase | WP_015127682 | 74 |
| ORF2 | hydrolase | missing | missing | 214 | missing | N/A | N/A | N/A | *Nostoc* sp. NIES-3756 | 2-hydroxy-3-keto-5-methylthiopentenyl-1-phosphate phosphatase | WP_067763881 | 87 |
| ORF3 | transferase fragment | missing | missing | 73 | missing | N/A | N/A | N/A | *Elaeis guineensis* | probable glutathione S-transferase parA | XP_010911024 | 25 |
| ORF1 | methyltransferase | missing | missing | missing | 289 | N/A | N/A | N/A | *Oscillatoria acuminata* | methylase | WP_015148146 | 52 |
| ORF2 | cytochrome P450 (oxidase) | missing | missing | missing | 461 | N/A | N/A | N/A | *Limnoraphis robusta* | cytochrome P450 | WP_046278736 | 66 |
